# ﻿Contributions of a small collection of terrestrial microsnails (Pupilloidea, Hypselostomatidae) from Myanmar with description of three new species

**DOI:** 10.3897/zookeys.1195.112112

**Published:** 2024-03-14

**Authors:** Piyoros Tongkerd, Ngwe Lwin, Barna Páll-Gergely, Ratmanee Chanabun, Arthit Pholyotha, Pongpun Prasankok, Teerapong Seesamut, Warut Siriwut, Ruttapon Srisonchai, Chirasak Sutcharit, Somsak Panha

**Affiliations:** 1 Animal Systematics Research Unit, Department of Biology, Faculty of Science, Chulalongkorn University, Bangkok 10330, Thailand; 2 Fauna and Flora International, No. 35, 3rd Floor, Shan Gone Condo, Myay Ni Gone Market Street, Sanchaung Township, Yangon, Myanmar; 3 Plant Protection Institute, Centre for Agricultural Research, ELKH, Herman Ottó út 15, 1022 Budapest, Hungary; 4 Program in Animal Science, Faculty of Agricultural Technology, Sakon Nakhon Rajabhat University, Sakon Nakhon 47000, Thailand; 5 Biodiversity and Utilization Research Unit, Center of Excellence in Modern Agriculture, Sakon Nakhon Rajabhat University, Sakon Nakhon 47000, Thailand; 6 School of Biology, Institute of Science, Suranaree University of Technology, Nakhon Ratchasima 30000, Thailand; 7 Department of Biology, Faculty of Science, Rangsit University, Pathum Thani 12000, Thailand; 8 Animal Systematics and Molecular Ecology Laboratory, Department of Biology, Faculty of Science, Mahidol University, Bangkok 10400, Thailand; 9 Department of Biology, Faculty of Science, Khon Kaen University, Khon Kaen 40002, Thailand; 10 Academy of Science, The Royal Society of Thailand, Dusit, Bangkok 10300, Thailand

**Keywords:** Conservation, endemism, FFI, limestone, systematics

## Abstract

Land snails were collected for the project ‘Conserving Myanmar’s Karst Biodiversity’ from the limestone karsts in Mon, Kayin, and Shan states and in the regions of Tanintharyi and Mandalay between 2015 and 2017, through cooperation with Fauna and Flora International (FFI) and the Forestry Department of Myanmar. Here, we report on a portion of the collection, and list 17 species from seven genera of the Hypselostomatidae microsnails. Three new species from two genera are described as *Bensonellataiyaiorum* Tongkerd & Panha, **sp. nov.**, *B.lophiodera* Tongkerd & Panha, **sp. nov.**, and *Gyliotrachelaaunglini* Tongkerd & Panha, **sp. nov.** All new species are known only from the type locality in Shan State (*Bensonella*) and Kayin State (*Gyliotrachela*). A new combination of *Acinolaemusdayanum* and three newly recorded species, namely *A.cryptidentatus*, *B.anguloobtusa* and *G.hungerfordiana* are discussed. The low morphological variability of the widely distributed *G.hungerfordiana* is discussed, and two species are proposed for formal synonymisation. Constituting the first records for Myanmar, five species of *Bensonella* and two species of *Acinolaemus* were collected.

## ﻿﻿Introduction

The Indo-Burma Region is globally recognised as a biodiversity hotspot that supports many species unique to Southeast Asia, and at the same time, it is one of the most threatened due to its high population density (Mittermeier et al. 2004; Tordoff et al. 2012; CEPF 2020). The limestone habitats are home to uniquely adapted plants and animals that have evolved in the special micro-habitat conditions existing there. Various families of land snails are associated with limestone habitats; one of these, Hypselostomatidae Zilch, 1959, possesses a trumpet shell shape and contains the world’s smallest land snails. Although some researchers consider this group a subfamily and others a distinct family (Schileyko 1998; Bouchet and Rocroi 2005; MolluscaBase 2023), we regard it as Hypselostomatidae because it lacks a broad-scale phylogeny and wish to maintain consistency with recent revisions (i.e., Páll-Gergely et al. 2015, 2020a, 2022).

Members of the family Hypselostomatidae are generally known as ‘microsnails’, possessing shells smaller than 5 mm (Panha and Burch 2005). They are widely distributed in Southeast Asia, southern China, Australia, and the Philippines, where they inhabit limestone-rich areas (Páll-Gergely et al. 2015, 2022). Studies of Burmese microsnails dates back to the mid-19^th^ to early 20^th^ century, during the British rule in Burma (1824–1948; now Myanmar) by the European naturalists (i.e., Gude 1914 and references therein). No new information records of new species were published in the following hundred years, until recently, when exploration of the hypselostomatid microsnails resumed. Prior to this study, only eleven hypselostomatid microsnails belonging to six genera were known from Myanmar. Among the five genera, *Clostophis* Benson, 1860 and *Angustopila*Jochum et al., 2014 have been systematically revised recently and are well-documented for Myanmar (Gude 1914; Páll-Gergely et al. 2020a, 2023; Gojšina et al. 2022; Páll-Gergely and Hunyadi 2022). Meanwhile the other genera, *Anauchen* Pilsbry, 1917, *Bensonella* Pilsbry & Vanatta, 1900, *Gyliotrachela* le Tomlin, 1930 and *Hypselostoma* Benson, 1856 have received little attention, with only a few reports (Gojšina et al. 2022; Páll-Gergely 2023a; Páll-Gergely and White 2023).

With the invitation from Fauna and Flora International (FFI) and the Forestry Department of Myanmar, we joined the ongoing ‘Conserving Myanmar’s Karst Biodiversity’ project to survey the land snails from the limestone karsts in the areas of Mon, Kayin, and Shan states and Tanintharyi and Mandalay regions between 2015 and 2017. The surveys have led to several systematic revisions and descriptions of new taxa, including the following: new genera and species of the limacoid and helicoid snails (see Páll-Gergely et al. 2020c; Pholyotha et al. 2020, 2022a, b; Sutcharit et al. 2020b), the carnivorous snails (see Páll-Gergely et al. 2020b; Sutcharit et al. 2020a; Man et al. 2022), the door snails (see Man et al. 2023), and the Cyclophoroidea (see Páll-Gergely et al. 2021; Tongkerd et al. 2023). These revisionary works, including the discovery of new taxa, have vastly improved the documented knowledge of land snails while confirming the high biodiversity within the limestone habitats of the lowland areas in the Salween River Basin as well as in the upland areas of the Shan Plateau of Myanmar. In sync with these studies, we specifically address the hypselostomatid microsnails, a large but overlooked fraction of Myanmar land snails. This present work aims to record and investigate all species of hypselostomatid microsnails known from Myanmar based on the literature and recent collections from our ongoing project with Fauna and Flora International.

## ﻿﻿Material and methods

**Figure 1.**
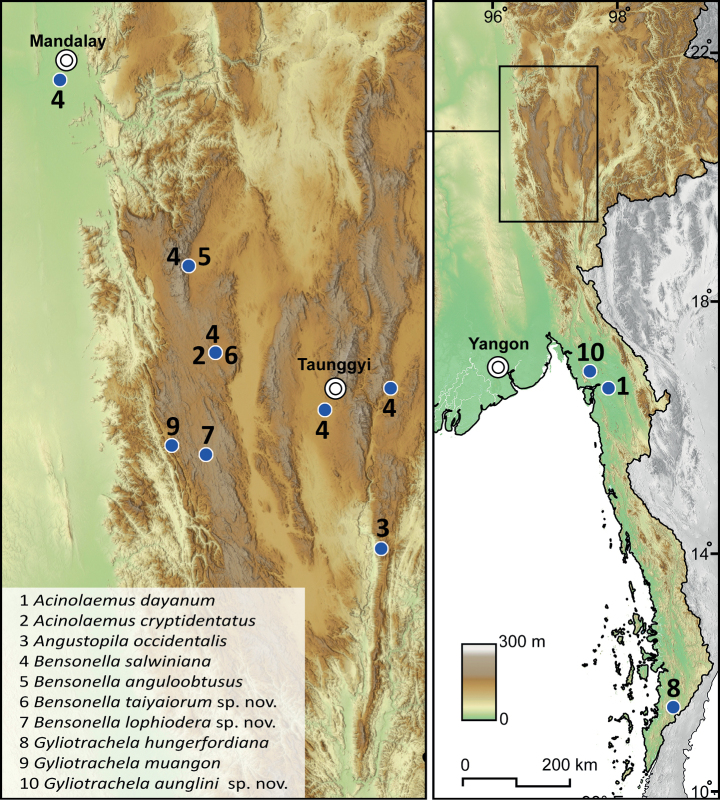
Approximate collecting localities of the hypselostomatid species from Myanmar examined in this study.

Microsnail samples were collected by hand while searching limestone walls and leaf litter (Fig. 1) in all accessible localities. Due to armed conflict in the country, specimen sampling and access was only allowed with special government permissions under an MOU (Memorandum of Understanding Agreement) between the Forest Department of the Ministry of Natural Resources and Environmental Conservation and Forestry, Myanmar and Fauna & Flora International, acknowledged by Letter No. 0092. Thus, only a small number of limestone sites in the Salween River Basin and Shan Hills could be explored between 2015 and 2017. The Salween karsts are located on the east slope of the Tenasserim Range, mainly in Mon and Kayin states and in the Tanintharyi Region. These regions have a typical tropical monsoon climate, with heavier rainfall usually occurring from May to November (Wang and Myint 2016). The Shan Hills, often called ‘Plateau Limestone’, are located in the east, mainly in the Shan and Kayah states and in the Mandalay Region. The area is bisected by the deep trench of the Salween River (Dreybrodt and Steiner 2015). The Shan Plateau has a mean elevation of approximately 1100 m and experiences a monsoon season between the end of May and October.

Shells were first soaked in a petri dish with water and detergent and then manually brushed of mud or dirt using fine painting brushes. The shells were air-dried, examined, and imaged by scanning electron microscopy (SEM; JEOL, JSM-6610 LV), and a Leica M205C microscope with a fusion optics stereo microscope and the Leica Application Suite Image System. Shell whorls were counted to the nearest quarter whorl. Shell measurements were taken from digital images by Cell’D Imaging Software (Olympus).

**Figure 2.**
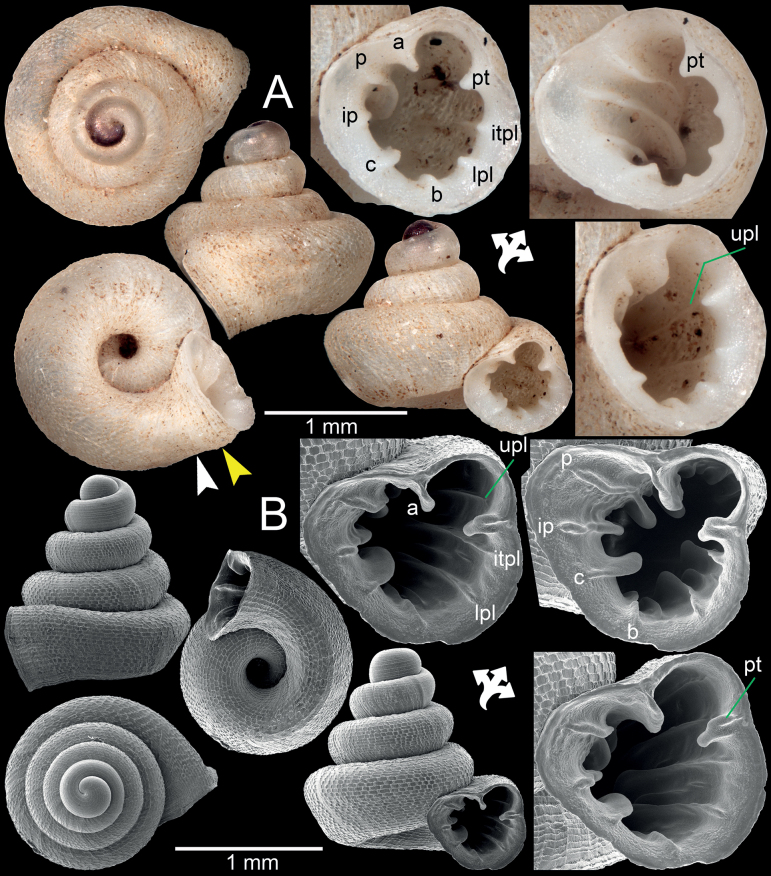
**A***Acinolaemusdayanum*, topotype specimen HA Collection from Mawlamyine Township, Mon State **B***Acinolaemuscryptidentatus*, specimen CUMZ 14413 from Taunggyi District, Shan State. The white arrow indicates constriction, the yellow arrow indicates a cervical crest. Abbreviations: **a**: angular lamella, **b**: basal plica, **c**: columellar lamella, **ip**: infraparietal lamella, **itpl**: interpalatal plica, **lpl**: lower palatal plica, **p**: parietal lamella, **pt**: palatal tubercle, **upl**: upper palatal plica.

The systematic relationships of the genera classified in the Hypselostomatidae are largely unclear. Several criteria are used to classify the representatives of this group. The nomenclature of apertural dentitions mostly follows Pilsbry (1948) and, regarding the palatal tubercle, Pilsbry (1918). To maintain consistency, the traditional genus-level identification of Pilsbry (1916–1918) has been used for *Hypselostoma* Benson, 1856 and *Gyliotrachela* le Tomlin, 1930. Meanwhile, *Angustopila*Jochum et al., 2014, *Bensonella* Pilsbry & Vanatta, 1900, and *Clostophis* Benson, 1860 have recently been re-defined, and several species have been systematically revised (Panha and Burch 2000; Páll-Gergely et al. 2022, 2023; Páll-Gergely and White 2023). The term ‘cervical crest’ refers to an additional swelling on the shell behind the aperture, as seen in lateral and umbilical views (Fig. 2A).

### ﻿Institutional abbreviations

**CUMZ**Chulalongkorn University Museum of Zoology (Bangkok, Thailand);

**HA** Collection András Hunyadi (Budapest, Hungary);

**HNHM**Hungarian Natural History Museum (Budapest, Hungary);

**IZCAS TM**National Zoological Museum of China, Institute of Zoology, Chinese Academy of Sciences (Beijing, China);

**MNHN**Muséum National ďHistoire Naturelle (Paris, France);

**NHM** The Natural History Museum (London, UK);

**NHMUK** When citing registered specimens;

**SMF**Senckenberg Forschungsinstitut und Naturmuseum (Frankfurt am Main, Germany);

**UMZC** University Museum of Zoology (Cambridge, United Kingdom).

## ﻿﻿Systematics

### ﻿﻿Superfamily Pupilloidea Turton, 1831

﻿**Family Hypselostomatidae Zilch, 1959**

#### ﻿﻿Genus Acinolaemus

Taxon classificationAnimaliaStylommatophoraHypselostomatidae

Thompson & Upatham, 1997

CF37E658-9402-5F8A-B7C8-D7154E059CAA

Acinolaemus Thompson & Upatham, 1997: 223, 224. Panha and Burch 2005: 39.

##### Type species.

*Acinolaemusptychochilus* Thompson & Upatham, 1997, by original designation.

##### Remarks.

The general shell characteristics of this genus, such as shell form, with or without tuba, and ascending to descending aperture, are similar to the other Southeast Asian hypselostomatid genera.

This genus has similar shell form, protoconch sculpture, tuba shape, and aperture opened (ascending or descending), making it congruent with those of other Southeast Asian hypselostomatid genera. Since only the shell morphology has been published so far, the systematic relationship with its confamilials remains unknown and needs further study. In the meantime, *Acinolaemus* can be distinguished from other genera in having a small size (height 1–2 mm), enlarged and conspicuous angular lamella, prominent posterior corner balloon-shaped by angular lamella and upper palatal plica. Moreover, the two species known from Myanmar are characterised by a teleoconch sculpture with a pattern of rectangular, malleated pitting, crossed by spiral and radial ridges.

Currently, ten nominal species of the genus are known to occur in Thailand and the Mekong Delta and in limestone areas of Cambodia and Vietnam (Changlom et al. 2019; MolluscaBase 2023). The two species reported here represent the first records of the genus *Acinolaemus* in Myanmar.

#### ﻿﻿ Acinolaemus dayanum

Taxon classificationAnimaliaStylommatophoraHypselostomatidae

(Stoliczka, 1871) comb. nov.

2CA29E2F-BF6C-5E4E-AC40-02448FB57B55

Figs 2A , 13A

Hypselostoma dayanum Stoliczka, 1871: 172, 173, pl. 7, fig. 2. Type locality: Damotha, prope Moulmein [Kayon Hill, Dhammasa Village, Mawlamyine District, Mon State]. Hanley and Theobald 1876: 59, pl. 147, fig. 10. Pfeiffer 1876: 488. Pfeiffer 1880: 344.
Boysidia (?) dayana. Pilsbry 1917: 205, 206, pl. 34, figs 5, 6.
*Pupa (Hypselostoma) dayana*. Nevill 1878: 193.
*Pupa (Hypselostoma) dayanum*. Gude 1914: 300, 301.

##### Material examined.

Dhammasa Cave (8 m), Mawlamyine centre NEE ca 26 km, Mon State, Myanmar (16.506715°N, 97.810763°E), leg. Otani, J.U., Okubo, K. and Hunyadi, A. 11 October 2018: HA Collection (11 shells; Figs 2A, 13A).

##### Description.

Shell turban shaped, low spire, ~ 3–4 whorls and whitish in colour. Shell height 1.2–1.3 mm and shell width 1.3–1.4 mm. Apex blunt; protoconch with fine spiral striae. Teleoconch sculptured with regularly spaced radial ribs, paralleled with strongly wrinkled and malleated pits; suture well impressed and deep. Last whorl shouldered and flattened below periphery. Peristome thickened and weakly expanded; constriction and cervical crest very weak; lip whitish. Aperture subquadrate with eight dentitions marking peristome. Parietal lamella large and long, deeper inside aperture with tall ridge; infraparietal lamella small with long and low rise. Angular lamella smaller and weaker than parietal lamella, long, low rise with slope somewhat bent. Palatal tubercle strongly developed with triangular shape. Upper-, inter-, and lower- palatal plicae small, connecting peristome, and continuous with thin and straight ridges deeper inside aperture. Basal plica small, tubercle-like. Columellar lamella strong, continuing deep inside aperture with undulated ridge. Umbilicus widely perforate, ~ 1/3 of shell width, rounded and deep.

##### Distribution.

Currently, this species is known only from the type locality, a limestone outcrop in Mon State, Myanmar.

##### Differential diagnosis.

*Acinolaemusdayanum* differs from all known *Bensonella* species in Myanmar and Thailand by its unique, turban shaped shell and teleoconch surface sculptured with malleated wrinkles and pits. In contrast, the four species from Myanmar and three species from Thailand, namely *B.tamphathai* (Panha & Burch, 2000), *B.nabhitabhatai* (Panha & Burch, 2002) and *B.pangmapaensis* (Panha & Burch, 2002) possess a conical shell and a teleoconch surface generally with conspicuous irregular growth lines and fine spiral striae (Panha and Burch 2000, 2002b).

Compared to the *Acinolaemus* species from Thailand, *A.dayanum* differs by having pitted sculpture on the shell surface, weaker spiral and radial striae, a larger parietal than angular lamella, and a shouldered last whorl. *Acinolaemusptychochilus* Thompson & Upatham, 1997 differs from *A.dayanum* by its rounded last whorl, strong tubercles on the peristomal lip, and the presence of supra- and subcolumellar lamellae. *Acinolaemuscolpodon* Thompson & Upatham, 1997 also has a rounded last whorl, possesses a hooked columellar lamella, and lacks an interpalatal plica, a basal plica and an infraparietal lamella. In addition, *A.sphinctinion* Thompson & Upatham, 1997 has a shouldered last whorl with blunt periphery, a short, weakly ascending tuba, and lacks the angular lamella, the parietal fold, the basal plica and the interpalatal plica (Thompson and Upatham 1997; Panha and Burch 2005).

Additionally, *A.dayanum* has a similar shell form and sculpture to the two *Acinolaemus* species recently described from northern Thailand. It differs from *A.cryptidentatus*Changlom et al., 2019, which has 4–5 whorls, strong spiral striae, and discontinuous infraparietal and angular lamellae. It is also distinct from *A.mueangonensis*Changlom et al., 2019 [corrected original spelling], which has 4–5 whorls, brownish shell colour, and a more prominent subcolumellar than columellar lamella.

##### Remarks.

The holotype (single shell mentioned in the original description) could not be located in the NHM collection, but we were able to examine recently collected topotypic shells. Originally, this species was classified in *Hypselostoma* and later reclassified into the genus *Boysidia* Ancey, 1881 (i.e., Stoliczka 1871; Pilsbry 1917). Based on the small size, the whitish shell and the strong teleoconch sculpture, we transfer *H.dayanum* into the genus *Acinolaemus*. In contrast, the genus *Bensonella* is characterised by a brownish shell with nearly smooth or finely, spirally striated shell (Páll-Gergely and White 2023), whereas *Hypselostoma* (at least the type species and the morphologically similar and geographically close species; see Gojšina et al. 2022 and Preece et al. 2022) and *Boysidia* are much larger, and have brownish, finely sculptured shells. Furthermore, *Hypselostoma* is defined on the basis of a detached body whorl and a concrescent angular and parietal lamellae, characters which do not appear in *A.dayanum*.

#### ﻿﻿ Acinolaemus cryptidentatus

Taxon classificationAnimaliaStylommatophoraHypselostomatidae

Changlom, Chan-ard & Dumrongrojwattana, 2019

AE9E8B58-A2CD-52CE-B382-45B7F37C7AEB

Figs 2B , 13B

Acinolaemus cryptidentatus Changlom et al., 2019: 158, 159, fig. 2a–f. Type locality: Tham Wua (Wua Cave), Mueang District, Mae Hong Son Province [Thailand].

##### Material examined.

Monastery, Ywangan Township, Taunggyi District, Shan State, Myanmar (locality code SH12; 21°13'43.3"N, 96°33'19.2"E): CUMZ 14413 (1 shell; Figs 2B, 13B).

##### Remarks.

This species was recently described from a limestone outcrop in northern Thailand that is ~ 200 km southeast of Taunggyi District, Shan State, Myanmar. This single specimen from Shan State slightly differs from the type specimen in having weak radial ridges and many weak interpalatal lamellae; whereas the type specimen possesses strong radial ridges and is without interpalatal lamellae. Further surveys should employ specialised sampling techniques (i.e., multiple series of sieves) that yield more specimens, which will clarify the identity of these tiny shells.

*Acinolaemuscryptidentatus* differs from *A.ptychochilus* and *A.mueangonensis* in having a shouldered last whorl and weak upper and lower palatal lamellae. In comparison, *A.ptychochilus* possesses a rounded last whorl, strong upper and lower palatal lamellae, and a strong infraparietal lamella. Meanwhile *A.mueangonensis* has a less-shouldered last whorl and lacks interpalatal lamellae (Thompson and Upatham 1997; Changlom et al. 2019).

#### ﻿﻿Genus Anauchen

Taxon classificationAnimaliaStylommatophoraHypselostomatidae

Pilsbry, 1917

FE6EFF6D-6A8E-551C-B422-9C5225C4CBD5

Anauchen Pilsbry, 1917: 174, 188. Panha and Burch 2005: 47.

##### Type species.

*Boysidiagereti* Bavay & Dautzenberg, 1904 (junior synonym of *Hypselostomarochebruni* Mabille, 1887; see Páll-Gergely 2023b).

#### ﻿﻿ Anauchen eotvosi

Taxon classificationAnimaliaStylommatophoraHypselostomatidae

Páll-Gergely, 2023

DFFC0951-A6F8-59DE-B0F3-9FB0BEEC5515

Fig. 13C

Anauchen eotvosi Páll-Gergely, 2023a: 452–454, fig. 1. Type locality: Shan-Siam Boundary.

##### Distribution.

This species was described based on specimens collected by Colonel Woodthorpe in 1894 or in 1895 from the ‘Shan-Siam Boundary’. Since the exact locality is unknown, it remains questionable that this species was originally collected from the area of present-day Laos or Myanmar.

#### ﻿﻿Genus Angustopila

Taxon classificationAnimaliaStylommatophoraHypselostomatidae

Jochum, Slapnik & Páll-Gergely, 2014

F41CFD18-AEE7-5F67-9EE2-DD8F73687221

Angustopila Jochum, Slapnik & Páll-Gergely, 2014 in Jochum et al. 2014: 26. Páll-Gergely et al. 2023: 17–24.

##### Type species.

*Systenostomatamlod* Panha & Burch, 2002, by original designation (Panha and Burch 2002a).

##### Remarks.

The genus was recently introduced to include the tiniest known land snails (Páll-Gergely et al. 2022). Species of this genus are characterised by tiny, colourless shells (typically less than 1.2 mm), 0–5 apertural barriers, and typically 10–20 spiral striae on the body whorl. Currently, the genus comprises more than 50 species that are mainly distributed in mainland Indochina (Laos, Myanmar, Thailand and Vietnam), with a few species recorded from southern China and a single species from India (Páll-Gergely et al. 2023). From Myanmar, two species are now recorded.

#### ﻿﻿ Angustopila occidentalis

Taxon classificationAnimaliaStylommatophoraHypselostomatidae

Páll-Gergely & Hunyadi, 2023

D0C5F334-569B-543B-A407-3671A8E17259

Figs 3 , 13D

Angustopila occidentalis Páll-Gergely & Hunyadi in Páll-Gergely et al. 2023: 118–122, figs 70–72. Type locality: Shan State, ca 6 km east from Hsihseng centre.

##### Material examined.

**Figure 3.**
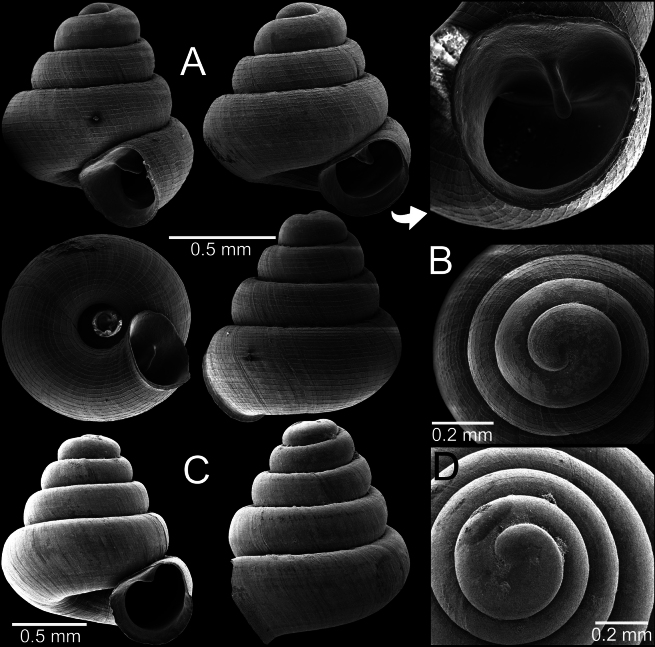
*Angustopilaoccidentalis*, specimen CUMZ 14389 from Taunggyi City, Shan State **A, B** specimen with four whorls and pronounced sculpture **C, D** specimen CUMZ 14390 with five whorls and weak or eroded sculpture.

Parpant area, Taunggyi City, Shan State, Myanmar (locality code: Sh8) 20°15'3.7"N, 97°14'23.9"E; 1159 m a.s.l.: CUMZ 14389 (1 shell; Fig. 3A, B), CUMZ 14390 (7 shells; Fig. 3C, D).

##### Distribution.

This species is known from several localities in Shan State, Myanmar (Páll-Gergely et al. 2023).

##### Remarks.

*Angustopilaoccidentalis* is uniquely recognised by a minute conical shell and whorls that are slightly shouldered and rounded on the periphery. The protoconch is uniquely radially striated (Fig. 3A, B), while all congeners known so far lack radial striation at this early developmental stage. Apertural dentition with one large parietal lamella.

In Myanmar, this species is widely distributed. The individuals examined herein were also collected from the limestone wall inside Parpant Cave, Shan State. These specimens agree well with the type specimen of this species (Fig. 13D) in having a medium-sized, low conical shell, one strong parietal lamella and lacking a palatal tooth.

#### ﻿﻿ Angustopila elevata

Taxon classificationAnimaliaStylommatophoraHypselostomatidae

(Thompson & Upatham, 1997)

44974E7B-BFCC-5A63-913C-EBCEFD98F98E

Fig. 13E

Systenostoma elevata Thompson & Upatham, 1997: 232, 233, figs 39–43. Type locality. Thailand, Chiang Mae Province [Chiang Mai Province], Doi Chiang Dao, 7 km west of Chiang Dao.
Angustopila elevata . Jochum et al. 2014: 27. Páll-Gergely et al. 2015: 33, fig. 11. Páll-Gergely et al. 2023: 27–32, figs 12, 13.
Angustopila subelevata Páll-Gergely et al., 2015: 39, fig. 4. Type locality: Jiaole Cun, Bama Xian, Hechi Shi, Guangxi, China. Páll-Gergely et al. 2017: 332, figs 1b, 2a–g, 7e, f.

##### Distribution.

This species is known from several localities in Shan State (Páll-Gergely et al. 2023).

##### Remarks.

Although this species was described from northern Thailand in Chiang Mai Province, subsequent revision by Páll-Gergely et al. (2023) has expanded its distribution to several localities in southern China, Laos, Myanmar, Vietnam and several additional localities in Thailand. It is considered one of the most widely distributed species, with a range spanning several hundred kilometres (Páll-Gergely et al. 2023).

The unique shell of this species is slightly concave-conical, bears a subquadrate aperture, lacking apertural dentitions, and shows a weakly elevated parietal wall.

#### ﻿﻿Genus Bensonella

Taxon classificationAnimaliaStylommatophoraHypselostomatidae

Pilsbry & Vanatta, 1900

F3D6C807-6527-5882-B74B-0D979D70DD2D

Bifidaria (Bensonella) Pilsbry & Vanatta, 1900: 591.
Boysidia (Bensonella) . Pilsbry 1917: 198.
Boysidia (Paraboysidia) Pilsbry, 1917: 174, 201.

##### Type species.

*Pupa plicidens* Benson, 1849, by original designation.

##### Remarks.

The generic status and diagnostic morphological characters of the type species were recently revised (Páll-Gergely and White 2023). The type species was believed to possess ‘hooked’ teeth, i.e., apertural barriers forming hooks that point outside of the aperture (Pilsbry 1917). Later, when specimens without hooked apertural barriers were found in collections, normal and hooked teeth were hypothesised as intraspecific variability (Budha and Backeljau 2017). Páll-Gergely and White (2023) showed that the ‘real’ *Bensonellaplicidens* was a species with normal (not hooked) barriers, while the Himalayan species with hooked teeth was an undescribed species (*Bensonellahooki* Páll-Gergely, 2023). Since the ‘hooked’ and ‘not hooked’ species are very similar in other shell characters, this trait could not be used as a diagnostic character for recognising *Bensonella*. Rather, this genus is diagnosed by a brownish shell with the last whorl attached to the penultimate whorl, and the presence of separate angular and parietal lamellae.

Since the type species of *Bensonella* and *Paraboysidia* (*Boysidiapaviei* Bavay & Dautzenberg, 1912) are very similar, the latter has been treated as a junior synonym of the former (Gittenberger et al. 2021; Páll-Gergely and White 2023).

#### ﻿﻿ Bensonella salwiniana

Taxon classificationAnimaliaStylommatophoraHypselostomatidae

(Theobald, 1871)

35DDA19D-812C-590B-8914-D6A9EB13387B

**Figure 4.**
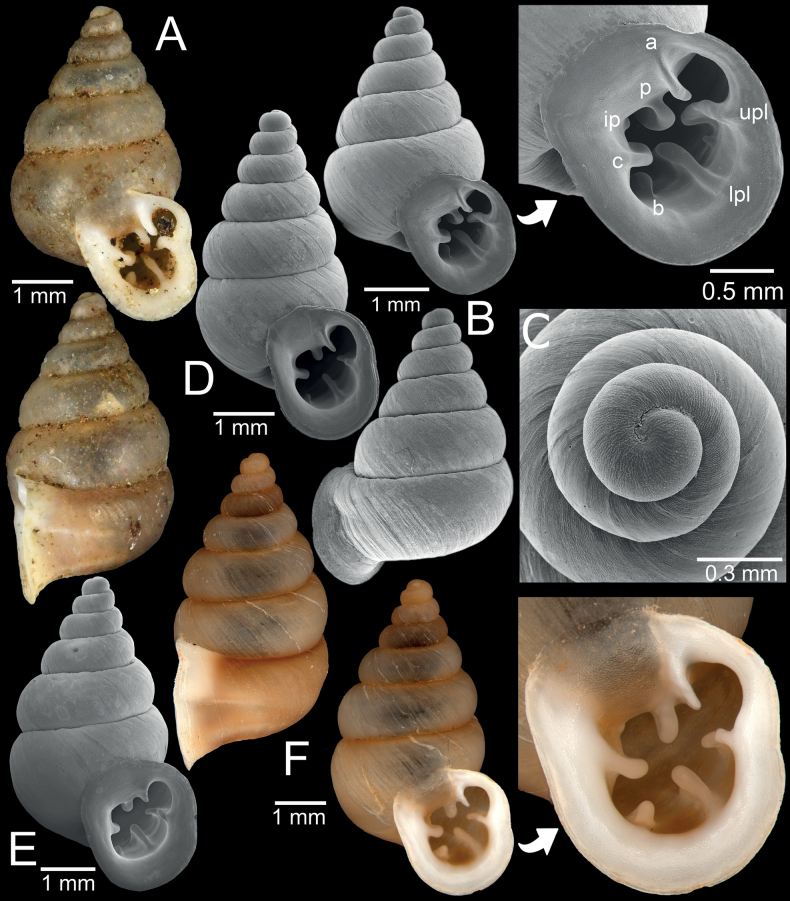
*Bensonellasalwiniana***A** specimen NHMUK 1912.4.16.66 from Burma **B, C** specimen CUMZ 14377 from Pyigyidagun Township, Mandalay Region **D** specimen CUMZ 14376 from Taunggyi District, Shan State **E** specimen CUMZ 14375 from Taunggyi District, Shan State **F** specimen CUMZ 14393 from Taunggyi District, Shan State. Abbreviations: **a**: angular lamella, **b**: basal plica, **c**: columellar lamella, **ip**: infraparietal lamella, **lpl**: lower palatal plica, **p**: parietal lamella, **upl**: upper palatal plica.

**Figure 5.**
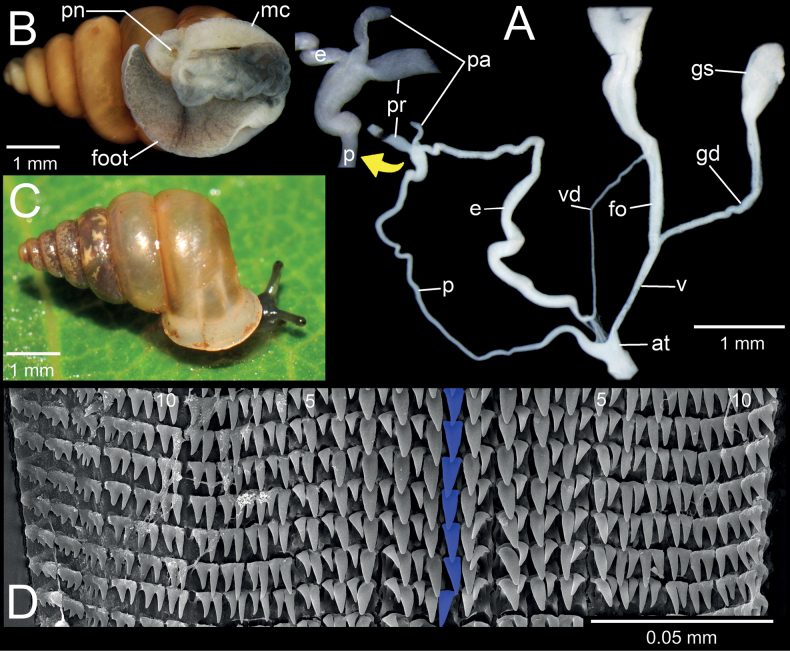
*Bensonellasalwiniana*, specimen CUMZ 14397 from Taunggyi District, Shan State **A** genitalia and a small inset of penial appendix **B** preserved specimen with mantle edge, body and foot **C** snail showing colour in life **D** radula morphology: blue highlighting indicates central teeth and numbers indicate tooth order from lateral to marginal end. Abbreviations: **at**: atrium, **e**: epiphallus, **fo**: free oviduct, **gd**: gametolytic duct, **gs**: gametolytic sac, **mc**: mantle collar; **p**: penis, **pa**: penial appendix, **pn**: pneumostome; **pr**: penial retractor muscle, **v**: vagina, **vd**: vas deferens.

**Figure 6.**
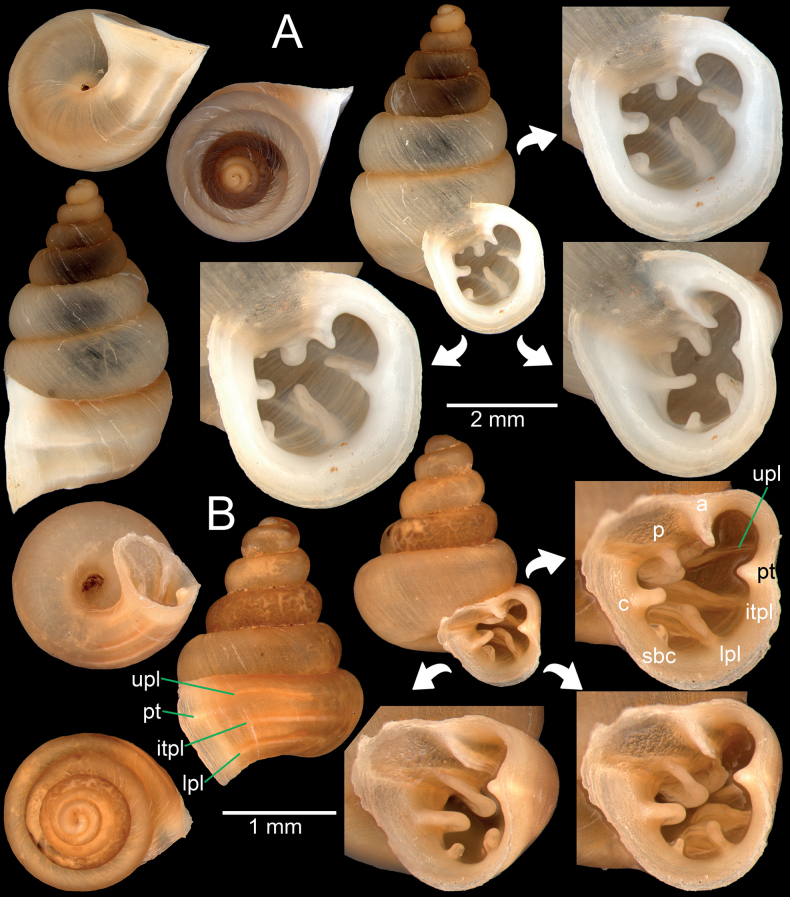
**A***Bensonellasalwiniana*, specimen CUMZ 14393 from Taunggyi District, Shan State **B***Bensonellaanguloobtusa*, specimen CUMZ 14401 from Taunggyi District, Shan State. Abbreviations: **a**: angular lamella, **c**: columellar lamella, **itpl**: interpalatal plica, **lpl**: lower palatal plica, **p**: parietal lamella, **pt**: palatal tubercle, **sbc**: subcolumellar lamella, **upl**: upper palatal plica.

Figs 4 , 5 , 6A , 13F

*Pupa salwiniana* Theobald, 1871: 400. Type locality: Shan States. Hanley and Theobald 1874: 40, pl. 100, fig. 9. Sowerby 1877: *Pupa* pl. 16, fig. 150. Pfeiffer 1877: 403.
*Pupa (Scopelophila) salwiniana*. Nevill 1877: 23.
*Pupa (Pupilla) salwiniana*. Pfeiffer 1880: 355.
*Pupa salwinieana* [sic]. Godwin-Austen 1888: 244.
Boysidia salwiniana . Gude 1914: 295, 296. Pilsbry 1917: 206–208, pl. 33, fig. 11.

##### Material examined.

Burma [Myanmar]: NHMUK 1912.4.16.66 (2 shells; Fig. 4A) ex. Beddome collection. Shan State: SMF 227428/2 (2 shells). Monastery, Ywangan Township, Taunggyi District, Shan State, Myanmar (locality code SH1; 21°13'43.3"N, 96°33'19.2"E): CUMZ 14375 (1 shell; Fig. 4E); CUMZ 14391 (2 shells); CUMZ 14392 (11 shells; measured); CUMZ 14393 (2 shells; Figs 4F, 6A, 13F). Dragon Rock, Pindaya Township, Taunggyi District, Shan State, Myanmar (locality code SH5; 20°55'31.5"N, 96°39'01.2"E): CUMZ 14376 (1 shell; Fig. 4D); CUMZ 14394 (4 shells). Blue Diamond Co., Ltd., Pyigyidagun Township, Mandalay Region, Myanmar (locality code MD1; 21°54'12.4"N, 96°04'38.8"E): CUMZ 14377 (1 shell; Fig. 4B, C); CUMZ 14395 (1 shell). Aik Kham Cave, Taunggyi District, Shan State, Myanmar (locality code SH10; 20°49'07.0"N, 97°13'42.0"E): CUMZ 14396 (22 shells). Montawa Cave, Taunggyi District, Shan State, Myanmar (locality code SH11; 20°45'15.8"N, 97°01'03.1"E): CUMZ 14397 (85 specimens in ethanol; Fig. 5).

##### Description.

Shell ovate-conical, high spire, yellowish brown, with 6–6½ convex whorls. Shell height 5.6–6.3 mm and shell width 3.3–3.7 mm. Apex blunt; protoconch ~ 1½ whorls, sculptured with radial wrinkles. Teleoconch with smooth, irregular growth lines; suture well impressed and deep. Last whorl large and rounded. Peristome thickened and broadly expanded; lip whitish. Aperture rounded-subquadrate with six or seven dentitions. Parietal lamella large, long, strong, broadly blunt, and located slightly deeper inside aperture; infraparietal lamella very small and sometimes absent. Angular lamella blunt and reaching peristomal lip. Palatal tubercle inconspicuous. Upper palatal plica long, contacting peristome and sometimes elevated in middle; lower palatal plica strong and large. Basal plica weak to strong nodule shape. Columellar lamella strong and large, tubercle-like. Umbilicus very narrowly perforate, rounded and deep.

***Genital system*.** Atrium short and slightly enlarged. Penis very long, thin tube and slightly enlarged at both ends; penial appendix short. Penial retractor muscle large and inserted between penis and epiphallus junction. Epiphallus slender tube, almost same length as penis, and ~ 2/3 of its length enlarged with similar diameter to free oviduct. Vas deferens very thin tube, connected between epiphallus and free oviduct, and held in position with thin connective tissue near epiphallus-vas deferens junction to atrium.

Vagina slender tube, short and ~ 1/3 of penis length. Gametolytic organ strongly developed; gametolytic duct slender tube, almost same length as vagina+free oviduct; gametolytic sac enlarged and bulbous. Free oviduct larger in diameter than vagina, and approximately same length as vagina.

***Animal*.** Preserved specimen with blackish to greyish reticulated skin, and mantle collar well-developed and whitish. Pneumostome wide and situated in the bay of angular lamella and upper palatal plicae. Foot short, holopodal, unipartite, and sole of foot blackish to greyish in colour. Living snails possess blackish tentacles: upper tentacles short and stout, and lower tentacles very short.

***Radula*.** Teeth arranged in nearly straight row with formula 15–(6, 5)–1–(5, 6)–11+. Central tooth unicuspid, with long triangular shape and pointed tip. Lateral teeth bicuspid, inner and outer cusps clearly separated at base, situated next to each other, and nearly aligned in transverse row. Inner and outer cusps long and triangular, and outer cusp comparatively smaller than inner cusp. Marginal teeth starting at approximately tooth number 5 or 6, inner and outer cusps of marginals joined at base. Innermost marginals bicuspid, similar to lateral teeth; outermost teeth multicuspid, cusps small with almost same size and shape, and situated on same base.

##### Distribution.

This species is known from several localities in northern Myanmar: Bhamo [Bhamo District, Kachin State]; Pingoung [Pingku Hills, Muse District, Shan State]; Shan Hills (Gude 1914; Pilsbry 1917). In this study, several specimens were also collected from Shan State and the Mandalay Region.

##### Differential diagnosis.

*Bensonellasalwiniana* differs from *Bensonella* species reported from Thailand and Laos in having a much larger (height 4–6 mm) and ovate conical shell, 6–7 whorls, inconspicuous palatal tubercle, and generally with six apertural dentitions. In contrast, *B.nabhitabhatai*, *B.tamphathai* and *B.pangmapaensis* tend to have smaller shells (height 2–4 mm), 4–5 whorls, a strong palatal tubercle, and generally bears 8–10 apertural dentitions (Panha and Burch 2000, 2002b).

For further comparison, the three species from Laos, namely *B.wangviangensis* (Panha & Tongkerd, 2003), *B.paralella* (Inkhavilay & Panha, 2016), and *B.anguloobtusa* (Inkhavilay & Panha, 2016) possess a smaller shell (height 1–3 mm), a strongly developed palatal tubercle and generally bears 3–4 whorls (Panha and Burch 2002b, 2005; Panha et al. 2003; Inkhavilay et al. 2016).

Although this species is very similar to *B.gittenbergeri* (Maassen, 2008) from Luang Namtha Province, Laos, in shell shape and size, it differs by having a lower palatal plica not extended to an expanded lip, a very narrow umbilicus, and an unextended palato-basal wall on the anterior side. In contrast, *B.gittenbergeri* possesses a long lower palatal plica that extends to an expanded lip, has a wider umbilicus, and its palato-basal wall is extended anteriorly (Maassen 2008; Inkhavilay et al. 2016).

*Bensonellasalwiniana* clearly differs from the two newly described species, *B.lophiodera* sp. nov. and *B.taiyaiorum* sp. nov., in having a larger shell (height 5–6 mm), and a broadly expanded peristome without cervical crest and lacking supra- and subcolumellar lamellae. Moreover, the latter two species possess smaller shells (height ~ 3 mm), a slightly expanded lip, and a conspicuous cervical crest. In addition, *B.taiyaiorum* sp. nov. has two upper palatal plicae, palatal tubercle strongly developed, supra-, subcolumellar lamellae and u-shaped plica present on the parietal wall, and a cervical crest far from the apertural lip. In contrast, *B.lophiodera* sp. nov. possesses a weakly expanded lip, subcolumellar lamella present and cervical crest close to apertural lip.

##### Remarks.

The species was described based on specimens received from F. Fedden (1839–1887). The specimens lacked a precise type locality, and only ‘Shan States’ was stated in the original description. Although the type specimen could not be located, several recently collected and historical specimens were examined. This species possesses strongly developed parietal and angular lamellae, and lacks a tuba, which are the diagnostic characters of *Bensonella*.

The examined individuals either possess or lack an infraparietal lamella.

#### ﻿﻿ Bensonella anguloobtusa

Taxon classificationAnimaliaStylommatophoraHypselostomatidae

(Inkhavilay & Panha, 2016)

E52B4268-DE2D-55D6-93BB-810824E2E96C

Figs 6B , 7A , 13G

Paraboysidia anguloobtusus Inkhavilay & Panha in Inkhavilay et al. 2016: 215–217, figs 2d–f, 4b. Type locality: Kao Rao Cave, Vieng Phouka District, Luang Namtha Province, Laos. Inkhavilay et al. 2019: 61, fig. 26f.

##### Material examined.

**Figure 7.**
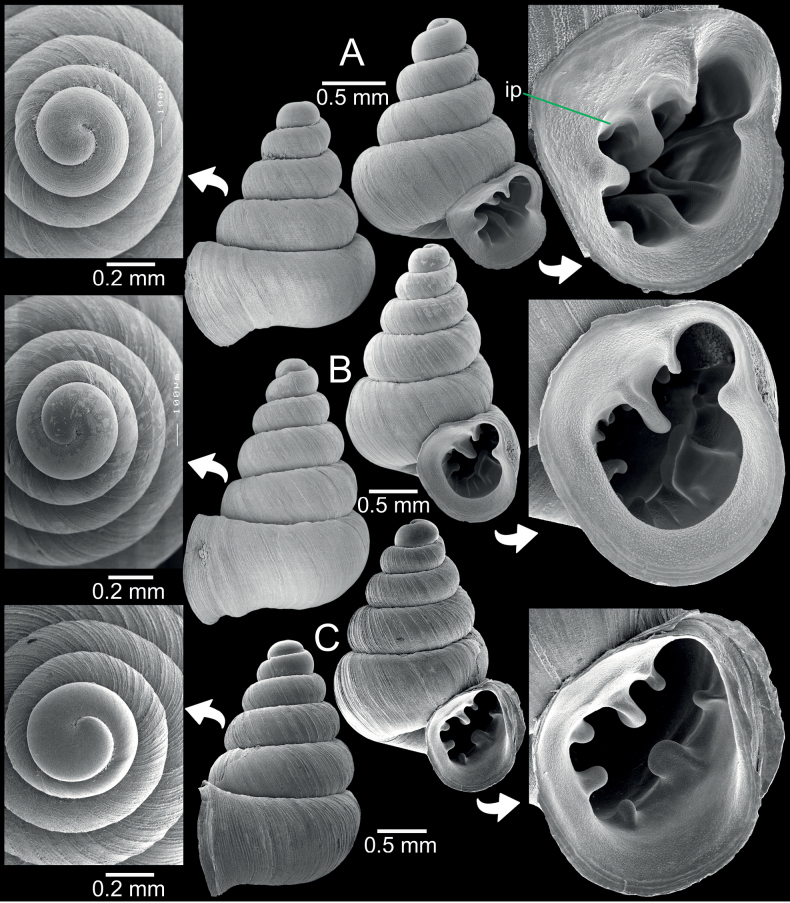
**A***Bensonellaanguloobtusa*, specimen CUMZ 14398 from Taunggyi District, Shan State **B***Bensonellataiyaiorum* sp. nov., holotype CUMZ 14380 from Taunggyi District, Shan State **C***Bensonellalophiodera* sp. nov., holotype CUMZ 14378 from Taunggyi District, Shan State. Abbreviation: **ip**: infraparietal lamella.

Monastery, Ywangan Township, Taunggyi District, Shan State, Myanmar (locality code SH2; 21°13'43.3"N, 96°33'19.2"E): CUMZ 14398 (1 shell; Fig. 7A); CUMZ 14399 (87 shells); CUMZ 14400 (12 shells; Fig. 13G; measured); CUMZ 14401 (1 shell; Fig. 6B).

##### Description.

Shell concave-conical, spire high, yellowish brownish in colour, and 4½–5 convex whorls. Shell height 1.7–2.1 mm and shell width 1.5–1.7 mm. Apex blunt; protoconch ~ 1½ whorls with wrinkled roundish pits and spiral ridges. Teleoconch with wrinkles, irregular growth lines, and very fine, dense, inconspicuous spiral striae; suture well impressed and deep. Last whorl convex, very slightly shouldered. Aperture subrectangular with eight or nine apertural barriers. Peristome thickened and little expanded, cervical crest absent; lip pale yellowish to brown. Parietal lamella strongly developed, robust, broadly blunt and starting deeper inside aperture than angular lamella; infraparietal lamella very small and sometimes absent. Angular lamella strong and low, as well as long and sinuous upon reaching peristome, increasing in height deeper inside aperture. Palatal tubercle strongly developed with triangular shape. Upper palatal plica long and very thin; interpalatal plicae and lower palatal plicae approximately same size, large and long, tubercle-like. Columellar lamella large, strong, and horizontal; subcolumellar lamella bears small, robust fold. Umbilicus perforate, ~ 1/4 of shell width, rounded and deep.

##### Distribution.

This species is currently known from the type locality in Luang Namtha Province, Laos. In the recent survey, individuals were collected from the limestone outcrops in Shan State, Myanmar.

##### Remarks.

This is the first record of the species in Myanmar. The new specimens were collected ~ 480 km away from the type locality. Therefore, more of these snails can be expected to be found in the northeastern part of Myanmar and the northern part of Thailand. The specimens from Shan State all agree well with the type specimen in both shell form and apertural dentition. However, only a very small infraparietal lamella is present in some individuals.

#### ﻿﻿ Bensonella taiyaiorum

Taxon classificationAnimaliaStylommatophoraHypselostomatidae

Tongkerd & Panha sp. nov.

2CDC58A5-71DF-5868-96B0-FF41EA56A00A

https://zoobank.org/E83B1A5F-A724-4BBF-9A7D-BB1604ECEFEF

Figs 7B , 8 , 13H

##### Type locality.

Dragon Rock, Pindaya Township, Taunggyi District, Shan State, Myanmar (locality code SH6; 20°55'31.5"N, 96°39'01.2"E; 1300 m a.s.l.).

##### Type material.

**Figure 8.**
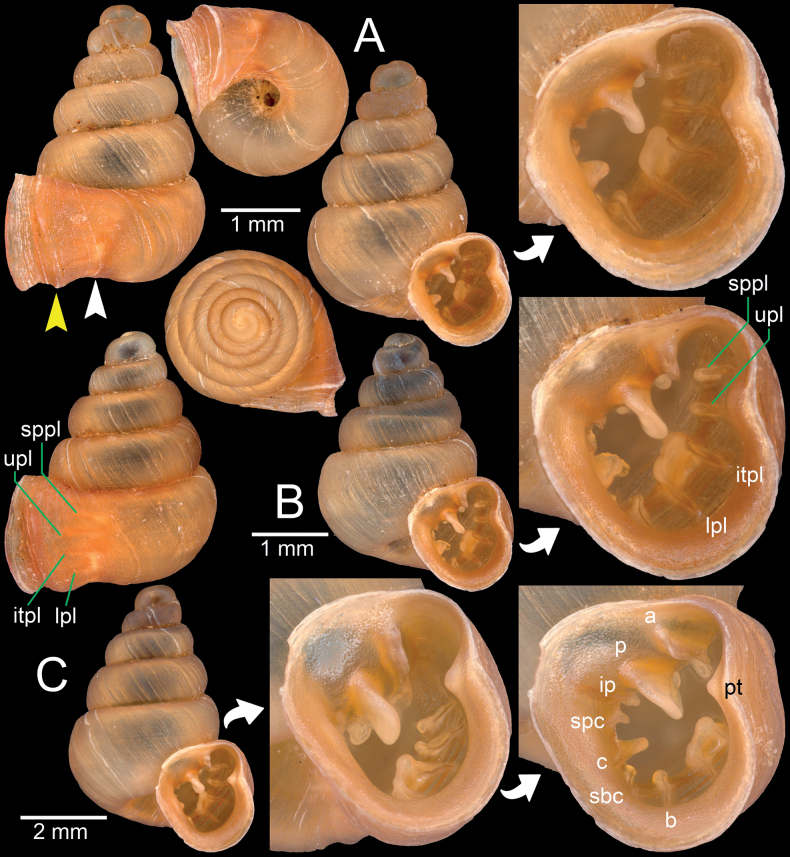
*Bensonellataiyaiorum* sp. nov., from Taunggyi District, Shan State **A–C** paratypes CUMZ 14403. The white arrow indicates constriction. The yellow arrow indicates a cervical crest. Abbreviations: **a**: angular lamella, **b**: basal plica, **c**: columellar lamella, **ip**: infraparietal lamella, **itpl**: interpalatal plica, **lpl**: lower palatal plica, **p**: parietal lamella, **pt**: palatal tubercle, **sbc**: subcolumellar lamella, **spc**: supracolumellar lamella, **sppl**: suprapalatal lamella, **upl**: upper palatal plica.

***Holotype***CUMZ 14380 (height 2.8 mm, width 2.1 mm; Fig. 7B). ***Paratypes***CUMZ 14381 (41 shells); CUMZ 14402 (12 shells; measured); CUMZ 14403 (3 shells; Figs 8, 13H), NHMUK 20230591 (3 shells), SMF 373019 (3 shells) all from the type locality.

##### Diagnosis.

Shell elongate-conical and yellowish brown in colour. Cervical crest situated far from peristome. Aperture with several hookless barriers (i.e., parietal, infraparietal, angular, columellar, supra- and sub-columellae, basal, and upper, inter and lower palatals), and interpalatal and lower palatal plicae forming u-shaped plica.

##### Etymology.

The specific name *taiyaiorum* is in recognition of the ‘Tai Yai’ or ‘Shan’, the largest ethnic group in Shan State, which is the type locality of this species.

##### Description.

Shell conical, yellowish brown to reddish brown and with 5¼–6 widely convex whorls. Shell height 2.5–2.9 mm and shell width 2.0–2.2 mm. Apex blunt; protoconch ~ 1½ whorls with wrinkles and very weak spiral ridges. Teleoconch with strong and irregular growth lines, sometimes white growth lines are conspicuous; suture impressed and deep. Last whorl large and rounded. Peristome thickened and slightly expanded; constriction weak; lip reddish brown. Cervical crest sharp and situated far from peristome. Aperture subrectangular and with many apertural dentitions. Parietal lamella strongly developed and consisting of two parts separated by an incision: outer part large, strong, with rectangular shape and reaching peristome; inner part blunt. Infraparietal lamella small and blunt. Angular lamella lower than parietal lamella: outer part short and reaching peristome; inner part more strongly elevated. Palatal tubercle strongly developed with triangular shape; two upper palatal plicae with strong folding; inter- and lower palatal large, equal in size and connected to form u-shaped plica. Basal plica small, low and tubercle-like. Columellar lamella large, strong and distinct in its horizontal alignment; one small supracolumellar lamella and one small subcolumellar lamella present. Umbilicus narrowly perforate, ~ 1/5 of shell width, rounded and deep.

##### Distribution.

This new species is known only from the type locality in Shan State. The snails live on limestone walls and can be found under leaf litter within rock crevices.

##### Differential diagnosis.

The new species can be distinguished from *Bensonella* reported from Thailand and Laos in having inter- and lower- palatal plicae connected, forming a u-shaped plica, and with prominent cervical crest. In contrast, the three species from Thailand differ by: *B.nabhitabhatai* has one upper palatal plica and no supracolumellar and infraparietal lamellae; *B.tamphathai* possesses fine spiral striae on the teleoconch, one upper palatal plica, and bears no supracolumellar lamellae; *B.pangmapaensis* has fine spiral striae on the teleoconch, and hooked palatal and basal plicae (Panha and Burch 2000, 2002b, 2005; Panha et al. 2003; Inkhavilay et al. 2016).

For further comparison, the three species from Laos can be distinguished by: *B.paralella* has one upper palatal plica, and no interpalatal plicae, supra- and sub- columellar lamellae; *B.anguloobtusa* has one upper palatal plica and no basal and supracolumellar lamellae; *B.wangviangensis* has a unique shape, smaller shell size (height 1–2 mm), four whorls, shouldered last whorl, and bears no basal plicae and columellar lamellae (Panha et al. 2003; Inkhavilay et al. 2016).

##### Remarks.

The two new species, *B.taiyaiorum* sp. nov. and *B.lophiodera* sp. nov., are the first *Bensonella* species to be described from Myanmar. The strongly developed and separated parietal and angular lamellae, and lack of a tuba are the main characters underscoring the assignment of these species to *Bensonella*.

Though the spiral striae on the protoconch are obscured under the light microscope, they are very faint but detectable under the SEM microscope. Of all the type series examined, no hooked-shaped dentitions were observed, while the u-shaped plicae are present in all specimens. A constriction on the palatal wall near the aperture and the cervical crest is possibly an important trait in response to resistance to desiccation.

#### ﻿﻿ Bensonella lophiodera

Taxon classificationAnimaliaStylommatophoraHypselostomatidae

Tongkerd & Panha sp. nov.

E517F338-19B3-5D2C-8852-B9AD358964C2

https://zoobank.org/068CD744-43A6-407A-9876-71B249D66C72

Figs 7C , 9 , 13I

##### Type locality.

Myinmati Cave, Kalaw Township, Taunggyi District, Shan State, Myanmar (locality code SH4; 20°35'24.6"N, 96°36'42.1"E; 1312 m a.s.l.).

##### Type material.

**Figure 9.**
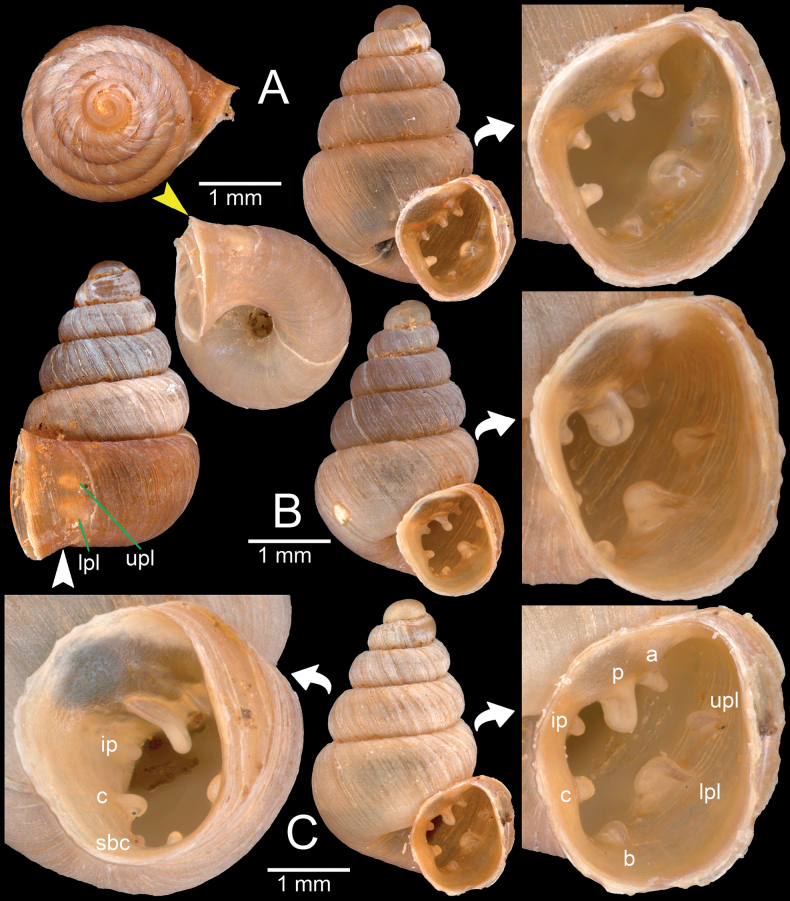
*Bensonellalophiodera* sp. nov., from Taunggyi District, Shan State **A–C** paratypes CUMZ 14405. The white arrow indicates constriction. The yellow arrow indicates a cervical crest. Abbreviations: **a**: angular lamella, **b**: basal plica, **c**: columellar lamella, **ip**: infraparietal lamella, **lpl**: lower palatal plica, **p**: parietal lamella, **sbc**: subcolumellar lamella, **upl**: upper palatal plica.

***Holotype***CUMZ 14378 (height 3.1 mm, width 1.8 mm; Fig. 7C). ***Paratypes***CUMZ 14379 (16 shells); CUMZ 14404 (12 shells; measured); CUMZ 14405 (3 shells; Figs 9, 13I), NHMUK 20230592 (3 shells), SMF 373020 (3 shells) all from the type locality.

##### Diagnosis.

Shell ovate-conical, high spired and pale yellowish brown colour. Cervical crest situated far from peristome. Lip not expanded. Aperture usually with seven barriers (parietal, infraparietal, angular, columellar, basal, and upper and lower palatals). Palatal tubercle is lacking.

##### Etymology.

The specific name *lophiodera* is a compound of the Greek words *lophia* meaning crest and *dere* meaning neck or throat. It refers to the prominent cervical crest on the palatal wall of the last whorl.

##### Description.

Shell ovate-conical, pale yellowish brown in colour, high spire and 5½–6 convex whorls. Shell height 2.7–3.2 mm and shell width 1.9–2.1 mm. Apex blunt; protoconch consisting of ~ 1½ whorls with wrinkles and weak spiral ridges. Teleoconch with somewhat strong and irregular growth lines; suture well impressed and deep. Last whorl large and rounded. Peristome slightly thickened and little expanded; constriction very weak; lip yellowish brown. Cervical crest sharp and situated close to peristome. Aperture subrectangular with seven or eight apertural barriers. Parietal lamella large, strongly developed, blunt, u-shaped from side view; infraparietal lamella small and knob shaped. Angular lamella weaker than parietal: outer part very low, weak and reaching peristome; inner part strong and tall. Palatal tubercle inconspicuous. Upper palatal plica small and low; lower palatal plica strong and tall, blunt. Basal plica small tubercle-like. Columellar lamella large and strong; sometimes an additional tiny subcolumellar lamella is present. Umbilicus narrowly perforate, ~ 1/5 of shell width, rounded and deep.

##### Distribution.

This species is currently known only from the type locality in Shan State. The living snails are found on limestone walls and under leaf litter in rock crevices.

##### Differential diagnosis.

This new species is similar to *B.salwiniana* in shell shape, number of apertural dentitions, and bears no palatal tubercle. However, *B.lophiodera* sp. nov. differs by having a strong cervical crest, and a very thin, fragile and unexpanded lip, while *B.salwiniana* shows no cervical crest, and has a thickened and expanded lip.

The new species is distinguishable from *Bensonella* reported from Thailand and Laos in having a weak palatal tubercle, a cervical crest, slightly expanded lip, no interpalatal plica, and apertural barriers are generally weak. Regarding the three species from Thailand, *B.nabhitabhatai* has one upper palatal plica, and lacks the basal plica and the infraparietal lamella; *B.tamphathai* possesses two interpalatal plicae, and its shell is sculptured with spiral striae; *B.pangmapaensis* has fine spiral striae and hooked palatal and basal plicae (Panha and Burch 2000, 2002b, 2005; Panha et al. 2003; Inkhavilay et al. 2016).

There are three *Bensonella* species known from Laos. Among them, *B.paralella* has an expanded lip and strongly prominent apertural dentitions; *B.anguloobtusa* has strong and sinuous apertural dentitions, and is sculptured with weak spiral striae; *B.wangviangensis* has a unique shell shape with small shell size (height 1–2 mm), a nearly closed sinulus, a shouldered last whorl, and lacks a basal plica and columellar lamella (Panha and Burch 2000, 2002b, 2005; Panha et al. 2003; Inkhavilay et al. 2016).

In addition, *B.lophiodera* sp. nov. differs from *B.taiyaiorum* sp. nov. in having eight apertural barriers, an inconspicuous palatal tubercle, one upper palatal plica, and lacking a supracolumellar lamella and interpalatal plica. In contrast, *B.taiyaiorum* sp. nov. possesses an expanded lip, has a strong palatal tubercle, two upper palatal plicae, and the inter- and lower- palatal plicae are connected and form a U-shaped plica.

##### Remarks.

The spiral striae on the protoconch are very faintly detectable under the SEM microscope. Among all the type series examined, no hooked-shaped dentitions were observed, while the apertural dentitions show minor variability in different degrees of thickness and sharpness. A clear constriction at the palatal wall near the aperture and a sharp cervical crest are present in all specimens.

#### ﻿﻿Genus Clostophis

Taxon classificationAnimaliaStylommatophoraHypselostomatidae

Benson, 1860

3AD78B1F-2BA2-5134-9043-3D383B26F079

Clostophis Benson, 1860: 95. Páll-Gergely et al. 2022: 419.
Montapiculus Panha & Burch, 2002c: 148.

##### Type species.

*Clostophissankeyi* Benson, 1860, by monotypy.

##### Remarks.

The genus was recently revised, and several new species have been introduced or transferred to this genus by Páll-Gergely et al. (2020a) and Páll-Gergely and Hunyadi (2022). The currently known 19 species of *Clostophis* are recognised and distributed from western Myanmar and Peninsular Malaysia to Thailand, Laos, northern Vietnam and southern China. The genus is characterised by tiny (1–2 mm) shells, colourless or whitish (but never brown) shells, mostly (but not always) detached last part of body whorl forming a free tube, shell surface with dense spiral striation, and possessing none or several apertural dentitions.

#### ﻿﻿ Clostophis sankeyi

Taxon classificationAnimaliaStylommatophoraHypselostomatidae

Benson, 1860

9857AF7F-0674-5288-BCC5-E6086D8DF6BF

Fig. 13J

Clostophis sankeyi Benson, 1860: 95, 96. Type locality: Farm Caves, prope [near] Moulmein.
Clostophis sankeyi . Páll-Gergely et al. 2020: 352, fig. 3a. Páll-Gergely and Hunyadi 2022: 419, figs 1, 2. Preece et al. 2022: 156, fig. 70e.

##### Distribution.

Known from the type locality ‘Farm Caves’ (= Dhammathat Cave, Mawlamyine, Mon State) and several localities from Mon State (Páll-Gergely and Hunyadi 2022). No specimen identified as this species was collected.

#### ﻿﻿ Clostophis thinbowguensis

Taxon classificationAnimaliaStylommatophoraHypselostomatidae

Páll-Gergely & Hunyadi, 2022

A7933933-B9FA-574C-96EA-A37C78753F63

Fig. 13K

Clostophis thinbowguensis Páll-Gergely & Hunyadi, 2022: 427, fig. 9. Type locality. Phayahran Camp, Thin Bow Gu Cave, Tanintharyi Region, Myanmar.

##### Distribution.

Known only from the type locality in Tanintharyi Region, Myanmar.

#### ﻿﻿Genus Gyliotrachela

Taxon classificationAnimaliaStylommatophoraHypselostomatidae

le Tomlin, 1930

6B2A119B-2DCB-5ED8-AFB7-95425A6D0956

Gyliotrachela le Tomlin, 1930: 24 [replacement name for Gyliauchen Pilsbry, 1917, non Nicoll 1915: Platyhelminthes, Trematodes]. Panha and Burch 2005: 63.

##### Type species.

*Hypselostomahungerfordianum* von Möllendorff, 1891, by typification of the replaced name.

##### Remarks.

This is a widely distributed and specious genus in Southeast Asia, currently comprising ~ 40 nominal species (MolluscaBase 2023).

#### ﻿﻿ Gyliotrachela bensonianum

Taxon classificationAnimaliaStylommatophoraHypselostomatidae

(Blanford, 1863)

032E262A-1B71-5658-BA8A-2072F2E9A2CA

Fig. 13L

Hypselostoma bensonianum Blanford, 1863: 326, 327. Type locality. Mya Leit Doung, Ava.
Hypselostoma bensonianum . Hanley and Theobald 1870: 4, pl. 8, fig. 2. Pfeiffer 1876: 488. Gude 1914: 299, 300.
*Pupa (Hypselostoma) bensoni*. Nevill 1878: 193.
Gyliauchen bensonianus . Pilsbry 1917: 211, pl. 37, figs 11, 12.
Gyliotrachela bensonianum . Gojšina et al. 2022: 138, figs 4a, 5a, b.

##### Distribution.

This species seems to be endemic to central Myanmar and is currently known only from the type locality, Mya Leit Doung, Ava [Myaleit mountains, Mandalay Region] (Gude 1914; Pilsbry 1917; Gojšina et al. 2022).

#### ﻿ Gyliotrachela hungerfordiana

Taxon classificationAnimaliaStylommatophoraHypselostomatidae

(von Möllendorff, 1891)

D418B341-51B2-5367-BEE8-8D2F39C7894C

Fig. 10 , 13M

Hypselostoma hungerfordianum von Möllendorff, 1891: 337, 338, pl. 30, fig. 7, 7a. Type locality: Bukit Pondong [Gunung Pondok, Padang Rengas, Perak, Malaysia]. Sykes 1902: 61.
Gyliauchen hungerfordianus . Pilsbry 1917: 212, pl. 36, figs 1–4.
Gyliotrachela hungerfordiana . Laidlaw 1933: 214. van Benthem Jutting 1949: 60. van Benthem Jutting 1950: 26. Zilch 1959: 164, fig. 563. van Benthem Jutting 1960: 14. Berry 1961: 101. Berry 1966: 12. Zilch 1984: 166, pl. 2, fig. 19. Davison 1995: 239. Schileyko 1998: 141, fig. 162. Schilthuizen et al. 1999: 283. Foon et al. 2017: 79, fig. 30b.
Gyliotrachela khaochongensis Panha, 1998: 53–56, fig. 2. Type locality: Khaochong Wildlife Sanctuary, Trang Province, Thailand. Panha and Burch 2005: 70, 71, fig. 61. syn. nov.
Gyliotrachela phoca Tongkerd & Panha in Tongkerd et al. 2013: 71–75, figs 5–7. Type locality: Bat cave near Klong Chak Waterfall, Lanta Yai Island, Lanta Islands National Park, Krabi Province, Thailand. syn. nov.

##### Material examined.

**Figure 10.**
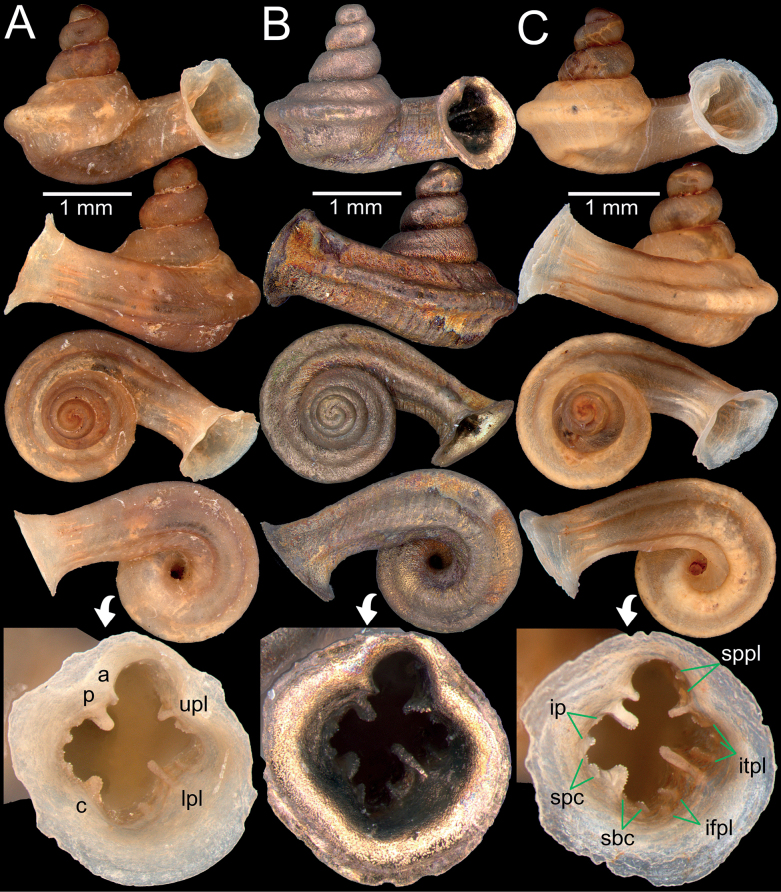
*Gyliotrachelahungerfordiana***A** topotype specimen NHMUK collection from Perak, Malaysia **B** paratype CUIZM, Ver 011 of *G.khaochongensis*, from Trang Province, Thailand **C** specimen CUMZ 14382 from Tanintharyi Region, Myanmar. Abbreviations: **a**: angular lamella, **c**: columellar lamella, **ip**: infraparietal lamella, **itpl**: interpalatal plica, **ifpl**: infrapalatal plica, **lpl**: lower palatal plica, **p**: parietal lamella, **sbc**: subcolumellar lamella, **spc**: supracolumellar lamella, **sppl**: suprapalatal lamella, **upl**: upper palatal plica.

Khaochong Wildlife Sanctuary, Trang Province, Thailand: Paratype CUMZ [CUIZM] Ver 011 (Fig. 10B). Bukit Pondok, Perak, Malaysia: NHMUK collection (2 shells: Figs 10A, 13M). Buddha Cave, Lenya city, Tanintharyi Region, Myanmar (locality code TN1; 11°13'46.2"N, 99°10'34.3"E): CUMZ 14382 (1 shell; Fig. 10C); CUMZ 14406 (61 shells); CUMZ 14407 (12 shells; measured).

##### Description.

Shell concave-conical, pale yellowish to brown in colour, moderate spire and 4–4½ widely convex whorls. Shell height 1.6–1.9 mm and shell width (including tuba) 2.5–2.9 mm. Apex blunt; protoconch ~ 1¾ whorls with wrinkled roundish pits. Teleoconch generally with very fine irregular growth lines and wrinkles; suture shallow. Last whorl angular with a prominent central keel with grooves above and below the keel. Tuba long and ~ 8–10 degrees angled upward compared to the shell axis. Peristome widely expanded; lip whitish to yellowish. Aperture roundly rectangular; aperture with many barriers. Parietal lamella large, strong, blunt, and located slightly deep inside aperture. Two small and weak infraparietal lamellae present. Angular lamella small, short, and reaching peristome. Upper palatal plica of approximately same size as angular lamella; very small suprapalatal plicae sometimes present. Lower palatal lamella tall and strong; very low and weak interpalatal plicae and infrapalatal plicae generally present. Columellar lamella strong and distinct; very low and weak supracolumellar and subcolumellar lamellae present. All dentitions generally covered with very fine spines on surface. Umbilicus widely perforate, ~ 1/3 of shell width, rounded, deep, and surrounded by blunt periumbilical keel.

##### Distribution.

This species has a wide distribution from southern Myanmar to southern Thailand and Peninsular Malaysia. Originally it was described from Perak and then subsequently reported from several localities in Peninsular Malaysia: Perlis, Pahang, Kelantan, Kedah, and Selangor states (von Möllendorff 1891; van Benthem Jutting 1949, 1950, 1960; Foon et al. 2017). This species was also reported in southern Thailand: Trang, Suratthani, Krabi, Patthalung, Songkla, and Satul provinces (Panha 1998; Panha and Burch 2005; Tongkerd et al. 2013). In Myanmar, this species recorded is known only from an isolated limestone karst in the Tanintharyi Region.

##### Differential diagnosis.

*Gyliotrachelahungerfordiana* differs from all other known *Gyliotrachela* species from Myanmar in having a thin shell, long and slender tuba, tall spire, and strong and curved keel on periphery. In contrast, *G.bensonianum* has a conical spire, short tuba, curved keel on last whorl, and apertural dentition without supra- and inter- palatal plicae or supra- and sub- columellar lamellae; *G.tianxingqiaoensis* and *G.muangon* possess angular last whorl, short tuba nearly adnate to last whorl, and with many small accessory plicae and lamellae (Gojšina et al. 2022).

##### Remarks.

*Gyliotrachelakhaochongensis* and *G.phoca* were described from southern Thailand; the type specimens look identical to the type and topotypic specimens of *G.hungerfordiana* in shell form, shell sculpture and apertural dentitions (Panha 1998; Tongkerd et al. 2013), with no significant differences. Therefore, they are considered here as junior synonyms of *G.hungerfordiana*.

#### ﻿﻿ Gyliotrachela tianxingqiaoensis

Taxon classificationAnimaliaStylommatophoraHypselostomatidae

(Luo, Chen & Zhang, 2000)

95DD6439-774D-55BA-B160-7F7759AA8A5B

Fig. 13N

Boysidia (Bensonella) tianxingqiaoensis Luo et al., 2000: 147, figs 1–4. Type locality. Tianxingqiao Town, Zhenning Bouyeizu Miaozu Zizhixian, Guizhou Province, China.
Gyliotrachela tianxingqiaoensis . Gojšina et al. 2022: 132–138, figs 1, 4b, 5c, d.

##### Distribution.

This species was originally described in Guizhou Province, China and subsequently reported from ‘Ava’ [Mandalay Region, Myanmar] based on the historical A.E. Salisbury collection (Luo et al. 2000; Gojšina et al. 2022).

#### ﻿﻿ Gyliotrachela muangon

Taxon classificationAnimaliaStylommatophoraHypselostomatidae

Panha & Burch, 2004

D053E9BE-285B-58D8-8A4B-E8C48DEB9638

Figs 11 , 13O

Gyliotrachela muangon Panha & Burch in Panha et al. 2004: 67, 68, fig. 7. Type locality. Muangon Cave, San Kam Pang District, Chiangmai Province, Thailand. Panha and Burch 2005: 76, 77, fig. 66. Gojšina et al. 2022: 138, fig. 6.

##### Material examined.

**Figure 11.**
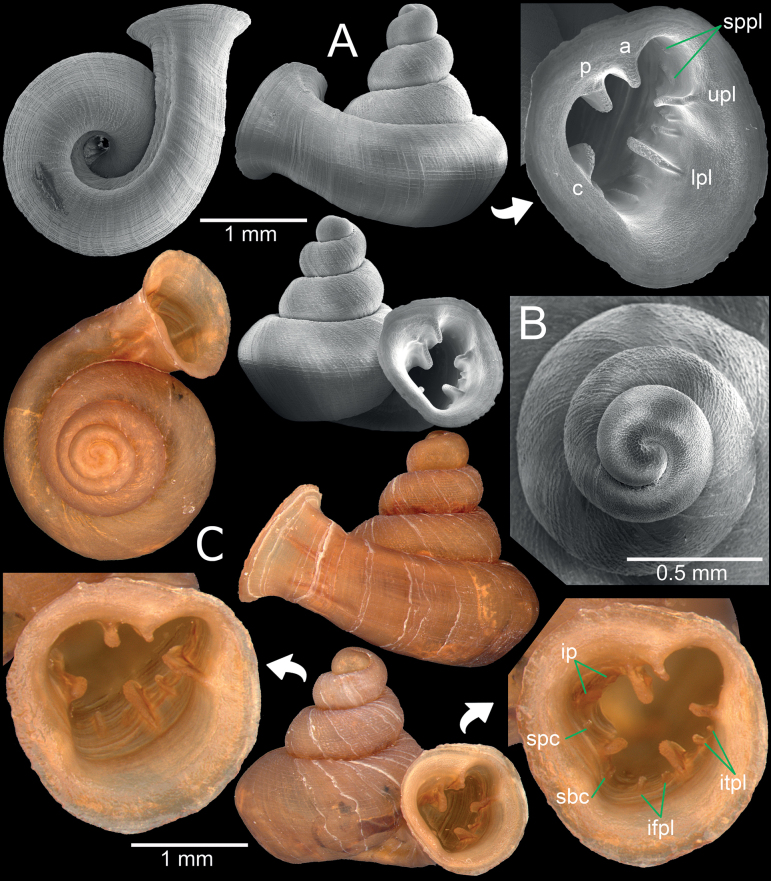
*Gyliotrachelamuangon* from Kalaw City, Shan State **A, B** specimen CUMZ 14385 from Kalaw City, Shan State, (**A**) shell and (**B**) protoconch **C** specimen CUMZ 14410 from Kalaw City, Shan State. Abbreviations: **a**: angular lamella, **c**: columellar lamella, **ip**: infraparietal lamella, **itpl**: interpalatal plica, **ifpl**: infrapalatal plica, **lpl**: lower palatal plica, **p**: parietal lamella, **sbc**: subcolumellar lamella, **spc**: supracolumellar lamella, **sppl**: suprapalatal lamella, **upl**: upper palatal plica.

Yum Cave, Kalaw City, Shan State, Myanmar (locality code KW1; 20°37'18.1"N, 96°29'8.3"E; 854 m a.s.l.): CUMZ 14385 (1 shell; Fig. 11A, B); CUMZ 14408 (6 shells); CUMZ 14409 (12 shells; measured); CUMZ 14410 (2 shells; Figs 11C, 13O).

##### Description.

Shell broadly ovate-conical, reddish brown in colour, moderate spire, and 4¾–5 widely convex whorls. Shell height 1.8–2.0 mm and shell width (including tuba) 2.5–2.9 mm. Appex blunt; protoconch 1¾ whorls, wrinkled with roundish pits. Teleoconch with wrinkles and irregular growth lines crossed by conspicuous spiral ridges throughout; suture well impressed and deep. Last whorl widely angular, tuba short and ~ 10–12 degrees angled upward. Peristome widely expanded; lip reddish brown. Aperture roundly triangular and many aperture dentitions. Parietal lamella large, tall, strong, blunt and slightly deep inside aperture. One or two minute infraparietal lamellae may be present. Angular lamella strong and reaching peristome. Upper palatal plica strong; sometimes very small suprapalatal plicae present. Lower palatal plica tall and strong; one or two small interpalatal plicae and infrapalatal plicae usually present. Columellar lamella very strong, distinct and horizontal; very low and weak supracolumellar and subcolumellar lamellae present. All dentitions generally with very fine spines on surface. Umbilicus perforate, ~ 1/4 of shell width, rounded, deep, and surrounded by blunt periumbilical keel.

##### Distribution.

This species was originally described from Chiangmai Province, northern Thailand (Panha et al. 2004). Later, a few specimens were collected from Hsi Hseng Township, Taunggyi District, Shan State (Gojšina et al. 2022). Recently, several specimens were collected from Kalaw Township, Taunggyi District, Shan State.

##### Differential diagnosis.

*Gyliotrachelamuangon* can be distinguished from *G.tianxingqiaoensis* from China and Myanmar, and *G.plesiolopa* Inkhavilay & Panha, 2016 from Laos by having weak spiral striae, five strong major lamellae and plicae (parietal, angular, columellar, and upper and lower palatals). In contrast, *G.tianxingqiaoensis* lacks spiral striation on the teleoconch, has a more elevated aperture, a narrower umbilicus and has more numerous teeth in the aperture; *G.plesiolopa* has a more expanded peristome, and a slightly weaker peripheral keel, which is also less upturned and flatter on the lower side (Inkhavilay et al. 2016; Gojšina et al. 2022).

The three species from Thailand (*G.saraburiensis* Panha & Burch in Burch et al. 2003, *G.muangon* and *G.cultura* Tanmuangpak & Dumrongrojwattana, 2022) are very similar to *G.muangon* in the turbinate shell form, the short and slightly descending tuba, the weakly angulated last whorl, the spiral striae on the teleoconch, and by having five major apertural dentitions. The significant distinction among them is mainly the number of accessory plicae and lamellae. Specifically, *G.muangon* possesses two infraparietals, two interpalatal and two infrapalatals, while *G.saraburiensis* has three infraparietals, three interpalatals and five infrapalatals, and *G.cultura* exhibits one infraparietal, one interpalatal and two infrapalatals (Burch et al. 2003; Panha et al. 2004; Panha and Burch 2005; Tanmuangpak and Dumrongrojwattana 2022).

##### Remarks.

The examined specimens from Myanmar show minor variability in terms of apertural dentition from the type specimen from Thailand. Two interpalatal plicae are observed in the Myanmar specimens, while the type specimen has only one interpalatal plica.

#### ﻿﻿ Gyliotrachela aunglini

Taxon classificationAnimaliaStylommatophoraHypselostomatidae

Tongkerd & Panha sp. nov.

F528EC79-C667-55AC-9FE6-834FBD02544A

https://zoobank.org/76E8570A-172A-4E2C-BB8A-7A1D898CD149

Figs 12 , 13P

##### Type locality.

Kaw Gon Cave, Hpa-An, Kayin State, Myanmar (locality code PA5; 16°49'22.2"N, 97°35'08.9"E).

##### Type material.

**Figure 12.**
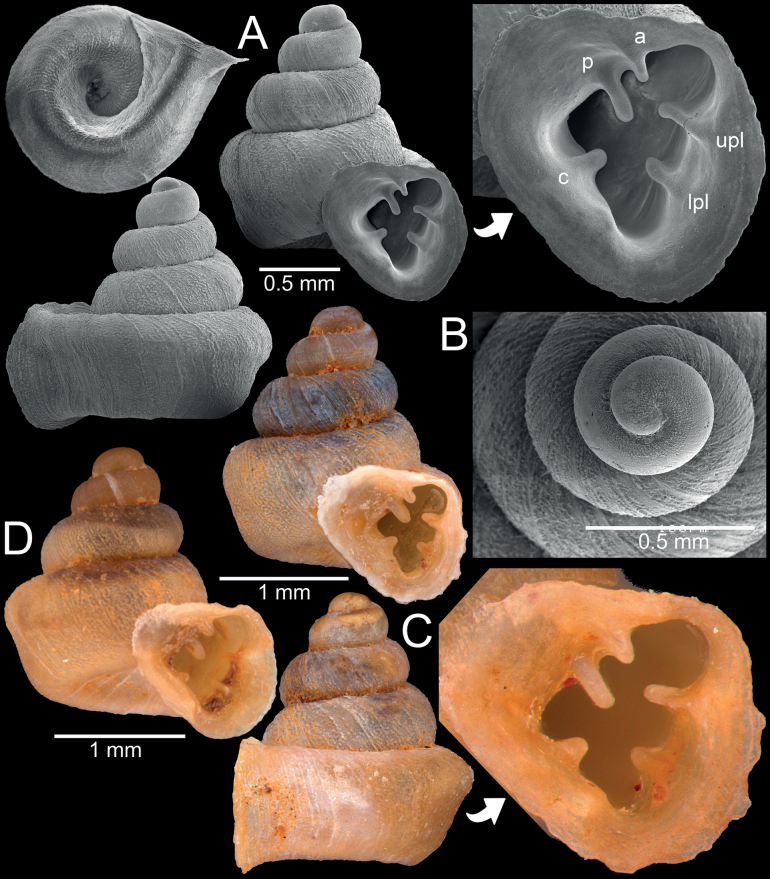
*Gyliotrachelaaunglini* sp. nov. from Hpa-An, Kayin State **A, B** holotype CUMZ 14383, (**A**) shell and (**B**) protoconch **C, D** paratypes CUMZ 14412 from the type locality. Abbreviations: **a**: angular lamella, **c**: columellar lamella, **lpl**: lower palatal plica, **p**: parietal lamella, **upl**: upper palatal plica.

**Figure 13.**
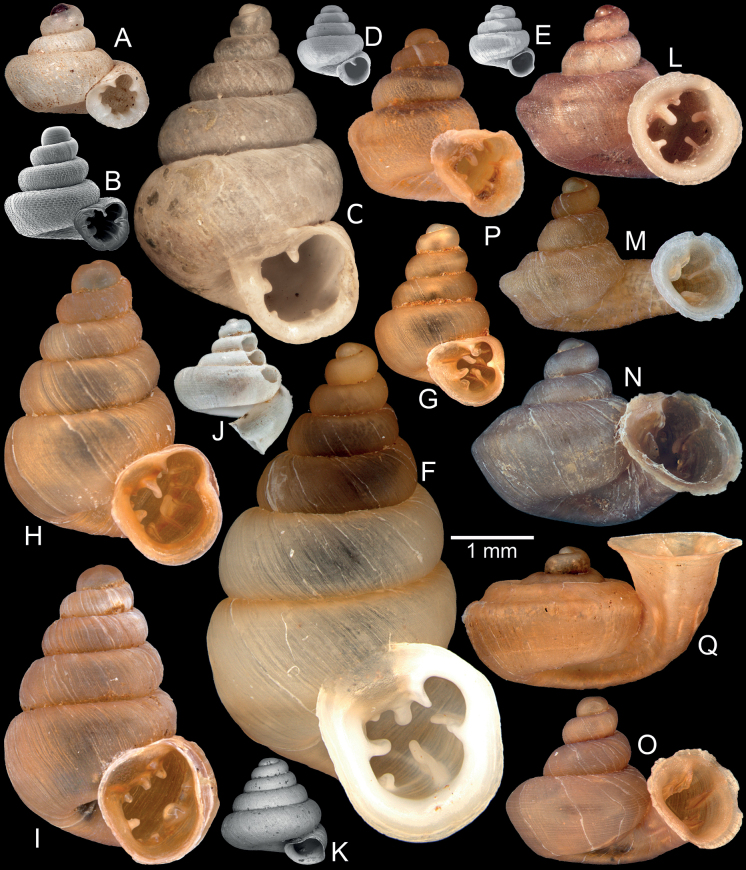
Synoptic view of representatives of hypselostomatid snails recorded from Myanmar **A***Acinolaemusdayanum*, specimen HA Collection **B***Acinolaemuscryptidentatus*, specimen CUMZ 14413 **C***Anaucheneotvosi*, holotype NHMUK 1903.7.1.1227.1 (after Páll-Gergely 2023a: fig. 1c) **D***Angustopilaoccidentalis*, holotype HNHM 103483 (after Páll-Gergely et al. 2023: fig. 70a) **E***Angustopilaelevata*, specimen HNHM 103484 (after Páll-Gergely et al. 2023: fig. 13a) **F***Bensonellasalwiniana*, specimen CUMZ 14393 **G***Bensonellaanguloobtusa*, specimen CUMZ 14400 **H***Bensonellataiyaiorum* sp. nov., paratype CUMZ 14403 **I***Bensonellalophiodera* sp. nov., paratype CUMZ 14405 **J***Clostophissankeyi*, holotype UMZC I.103320 (after Preece et al. 2022: fig. 70e) **K***Clostophisthinbowguensis*, holotype MNHN-IM-2000-38057 (after Páll-Gergely et al. 2022: fig. 9c) **L***Gyliotrachelabensonianum*, syntype NHMUK 20191141 (after Gojšina et al. 2022: fig. 4a) **M***Gyliotrachelahungerfordiana*, specimen NHMUK collection from Perak, Malaysia **N***Gyliotrachelatianxingqiaoensis*, holotype IZCAS TM 025075 (after Gojšina et al. 2022: fig. 3a) **O***Gyliotrachelamuangon*, specimen CUMZ 14410 **P***Gyliotrachelaaunglini* sp. nov., paratype CUMZ 14412 **Q***Hypselostomatubiferum*, specimen NHMUK 1888.12.4.17–22 ex. Theobald collection from Tonduong, Burma.

***Holotype***CUMZ 14383 (height 1.8 mm, width 1.6 mm; Fig. 12A, B). ***Paratypes***CUMZ 14384 (2 shells); CUMZ 14411 (16 shells; measured); CUMZ 14412 (2 shell; Figs 12C, D, 13P), NHMUK 20230593 (2 shells), SMF 373021 (2 shells) all from the type locality.

##### Diagnosis.

Shell ovate-conical with shouldered and flat-sided last whorl, aperture not detached from penultimate whorl; shell surface irregularly wrinkled, colour pale reddish brown; aperture roundly triangular, with five or six apertural dentitions (angular, parietal, upper palatal, lower palatal and columellar).

##### Etymology.

The specific name *aunglini* is named after Mr. Aung Lin, the FFI coordinator, who took care of the survey team and arranged the limestone survey trip in Myanmar between 2015 and 2016.

##### Description.

Shell broadly ovate-conical, pale reddish brown in colour, moderate spire, and 4½–5 widely convex whorls. Shell height 1.6–1.8 mm and shell width 1.6–1.8 mm. Apex blunt; protoconch with 1¾ whorls, wrinkled, with roundish pits and weak spiral ridges. Teleoconch with narrowly spaced radial growth lines, parallel with very strong, irregular wrinkles; suture well impressed. Last whorl bluntly shouldered and flat-sided with shallow groove below shoulder. Tuba absent; peristome adnate and widely expanded; lip whitish to yellowish. Aperture roundly triangular and with five dentitions. Parietal lamella large, strong, blunt and located somewhat deeper inside aperture than angular lamella. Angular lamella strong and reaching peristome. Upper palatal plica strong, tall and approximately same size as parietal lamella; lower palatal plica strong and lower than upper palatal plica. Columellar lamella strong, distinct and pointing slightly upwards. Umbilicus widely perforate, ~ 1/3 of shell width, rounded, shallow, and surrounded by curved periumbilical keel.

##### Distribution.

This new species is currently known only from the type locality in Kayin State. This snail lives on the limestone walls and under leaf litter.

##### Differential diagnosis.

*Gyliotrachelaaunglini* sp. nov. differs from *G.bensonianum* and *G.tianxingqiaoensis* from Myanmar in having high spire, peristome adnate to preceding whorl, shouldered last whorl, with very weak spiral striae on protoconch, and only five apertural dentitions (parietal, angular, upper- and lower- palatal, and columellar). In contrast, the other two species possess a depressed spire, a short and slightly ascending tuba, and an angular last whorl. Additionally, *G.bensonianum* has a spirally striated teleoconch, and it has a basal plica and infraparietal lamella; *G.tianxingqiaoensis* possesses numerous small accessory plicae and lamellae (Gojšina et al. 2022).

This new species can be distinguished from *G.saraburiensis*, *G.muangon*, and *G.cultura* (all known from Thailand) by having no tuba (i.e., peristome adnate to preceding whorl), possessing a shouldered last whorl, strong wrinkles on the shell surface and bearing only five to six major dentitions. In contrast, the three species from Thailand tend to have a short and slightly ascending tuba, an angular to weakly angular last whorl, spiral striae on the teleoconch, and numerous accessory plicae and lamellae inside the aperture (Burch et al. 2003; Panha et al. 2004; Panha and Burch 2005; Tanmuangpak and Dumrongrojwattana 2022).

Several *Gyliotrachela* species from southern Thailand are similar to the new species by having very short tuba, and few apertural dentitions. *Gyliotrachelaaunglini* sp. nov. differs from *G.transitans* (von Möllendorff, 1894) in the short and slightly descending tuba, the angular last whorl, and the apertural dentition with supracolumellar lamella and a basal plica; *G.tarutao* (Panha & Burch, 2002) has an elevated spire, an angular last whorl, a very short tuba, and the apertural dentition is characterised by a subcolumellar lamella, an infraparietal plica and a basal plica (von Möllendorff 1894; Panha and Burch 2002b, 2005). The new species differs from *G.adela* Thompson & Upatham, 1997 in the depressed spire, the short and descending tuba, with prominent spiral striae on the teleoconch, and apertural dentition with a basal plica (Thompson and Upatham 1997).

Further comparison can be made with species from Peninsular Malaysia that have a very short tuba and few apertural dentitions. *Gyliotrachelaaunglini* sp. nov. differs from *G.emergens* van Benthem Jutting, 1950 and *G.modesta* van Benthem Jutting, 1950 in having an elevated spire, short and slightly descending tuba and an angular or weakly angular last whorl. In addition, the apertural dentition of the former species includes a basal plica, and supra- and sub- columellar lamellae, while the latter species has a supracolumellar lamella and a weak infrapalatal plica. Additionally, this new species differs from *G.troglodytes* van Benthem Jutting, 1950 in the depressed spire, the very short and descending tuba, the angular last whorl, the apertural dentition with infraparietal and weak angular lamellae, and the strongly wrinkled shell surface (van Benthem Jutting 1950).

##### Remarks.

The material examined here shows minor variability in terms of shell size, but the key morphological characters, shell sculpture and apertural dentition, are reliable.

#### ﻿﻿Genus Hypselostoma

Taxon classificationAnimaliaStylommatophoraHypselostomatidae

Benson, 1856

64C763B0-2921-57BD-8B8A-5B5927D35723

Tanystoma Benson, 1856a: 130 [non de Motschulsky 1845: Coleoptera, Carabidae].
Hypselostoma Benson, 1856b: 342 [new replacement name]. Panha and Burch 2005: 87.

##### Type species.

*Tanystomatubiferum* Benson, 1856a, by monotypy.

##### Remarks.

So far, only one species has been reported from Myanmar.

#### ﻿﻿ Hypselostoma tubiferum

Taxon classificationAnimaliaStylommatophoraHypselostomatidae

(Benson, 1856)

84F6251A-9EEF-59A5-B48C-1EEFD7FA9DE9

Fig. 13Q

Tanystoma tubiferum Benson, 1856a: 130. Type locality: Thyet-Mio [Thayet District, Magway Division].
Hypselostoma tubiferum . Pfeiffer 1859: 325. Pfeiffer 1860: 130, pl. 36, figs 1–4. Blanford 1863: 326. Hanley and Theobald 1870: 4, pl. 8, fig. 3. Stoliczka 1871: 173, pl. 7, fig. 1. Pfeiffer 1876: 488. Gude 1914: 298, 299. Pilsbry 1917: 178, 179, pl. 31, figs 1–5. Gojšina et al. 2022: 138, fig. 7. Preece et al. 2022: 155, 156, fig. 70d.
*Pupa (Hypselostoma) tubifera*. Nevill 1878: 193.

##### Distribution.

So far, new specimens have yet to be collected, and only the historical museum collection was available for study. This species is known only from certain localities in central Myanmar: Thyet Mio [Thayet District, Magway Division]; Mya Leit Doung [Myaleit mountains, Mandalay Region]; Tsagyen Hills [Sagaing Hills, Sagaing Region]; Pegu and Henzada [Bago Region and Hinthada District, Ayeyarwady Region] (Gude 1914; Pilsbry 1917; Gojšina et al. 2022).

## ﻿﻿Discussion

This inventory updates previous work and provides a comprehensive record of the hypselostomatid snail fauna of Myanmar. Before the 20^th^ century, the record was compiled by Gude (1914), including five species belonging to the genera *Boysidia* and *Hypselostoma*. Currently, 17 species are known to occur in Myanmar. Among these, seven species, namely *Anaucheneotvosi*, *Angustopilaelevata*, *Clostophissankeyi*, *Clostophisthinbowguensis*, *Gyliotrachelabensonianum*, *Gyliotrachelatianxingqiaoensis*, and *Hypselostomatubiferum* are listed herein based solely on records in the literature (i.e., Gude 1914; Gojšina et al. 2022; Páll-Gergely and Hunyadi 2022; Páll-Gergely 2023a; Páll-Gergely et al. 2023). The synoptic views of the type or authenticated specimens of those species are provided for baseline comparison and identification. Additionally, three new species have been described, a new combination (*Acinolaemusdayanum*) was proposed, and three species are redescribed. Finally, three species (*Acinolaemuscryptidentatus*, *Bensonellaanguloobtusa*, *G.hungerfordiana*) are newly recorded for Myanmar.

*Gyliotrachela*, a genus which exhibits a wide range of morphological variability among the Hypselostomatidae, is represented by five species. The redescription of the type species, *G.hungerfordiana*, described initially from Peninsula Malaysia, together with ‘*G.khaochongensis*’ and ‘*G.phoca*’ from southern Thailand, are formally synonymised. Based on the recent collection from Myanmar, *G.hungerfordiana* and *G.muangon* appear to have relatively wide ranges of distribution; specimens of the former were collected 750 km from the type locality in Thailand, and specimens of the latter were found 250–350 km from the type locality. However, Burmese samples of both species exhibited some morphological variation, especially in terms of their ‘additional’ or ‘supplementary’ apertural dentition (i.e., tiny plicae between main plicae), although this does not appear useful in distinguishing species. The apparently low morphological variability of these widely distributed species may reflect that their broad distributions are due to recent dispersion events, or it may show convergence of the diagnostic value of shell traits. Additional new material and genetic data could help to address this question.

*Bensonella* (now united with *Paraboysidia*) and *Acinolaemus* have now been reported for the first time in Myanmar, and are represented by five and two nominal species, respectively. One of the little-known species, *A.dayanum* originally described from limestone hills in the Salween River Basin, has a shell that is morphologically unique and somewhat intermediate in that it demonstrates certain shell characters of other species. For instance, it shares a large angular lamella and wide umbilicus with *Acinolaemus*, and shares a cervical crest on the palatal wall behind the lip and three lamellae on the parietal wall with *Bensonella*; at the same time, the malleated pits of its shell sculpture seem to be a unique character, only shared with the probably closely related *Acinolaemuscryptidentatus*. However, the relationship between *Acinolaemus* and *Bensonella* could not be interpreted here, since a molecular phylogeny of some hypselostomatid taxa indicated that shell morphology, especially aperture dentition, can only be used with caution as diagnostic characters. Instead, shell sculpture is more taxonomically significant (Tongkerd et al. 2004). However, this study has attempted to present such differentiation as a baseline regarding shell morphology for future studies, especially concerning the phylogenetic interpretation of these genera.

Myanmar has the largest total karst area among the Indochinese countries and contains extensive limestone areas, i.e., Shan Plateau and Andaman coastal area (Day and Urich 2000; Gunn 2004; Clements et al. 2006). However, only a handful of studies concerning Myanmar’s hypselostomatid snails have been published, and their known diversity is low compared to nearby countries. Therefore, extensive surveys covering a range of limestone habitats across different elevations, latitudes and karstic regions could be expected to uncover more cryptic taxa. Most hypselostomatids are smaller than 5 mm and prefer specific microhabitats such as limestone walls, caves, and litter. Therefore, we suggest using focused collecting techniques and procedures such as soil sampling from various habitat types and sieving via graduated sieves (Nekola and Coles 2010; Neubert and Bouchet 2015; Páll-Gergely et al. 2023).

## Supplementary Material

XML Treatment for Acinolaemus

XML Treatment for Acinolaemus dayanum

XML Treatment for Acinolaemus cryptidentatus

XML Treatment for Anauchen

XML Treatment for Anauchen eotvosi

XML Treatment for Angustopila

XML Treatment for Angustopila occidentalis

XML Treatment for Angustopila elevata

XML Treatment for Bensonella

XML Treatment for Bensonella salwiniana

XML Treatment for Bensonella anguloobtusa

XML Treatment for Bensonella taiyaiorum

XML Treatment for Bensonella lophiodera

XML Treatment for Clostophis

XML Treatment for Clostophis sankeyi

XML Treatment for Clostophis thinbowguensis

XML Treatment for Gyliotrachela

XML Treatment for Gyliotrachela bensonianum

XML Treatment for Gyliotrachela hungerfordiana

XML Treatment for Gyliotrachela tianxingqiaoensis

XML Treatment for Gyliotrachela muangon

XML Treatment for Gyliotrachela aunglini

XML Treatment for Hypselostoma

XML Treatment for Hypselostoma tubiferum

## ﻿Additional information

### Author ORCIDs

Piyoros Tongkerd https://orcid.org/0000-0001-9221-9293

Ngwe Lwin https://orcid.org/0000-0002-4394-3741

Barna Páll-Gergely https://orcid.org/0000-0002-6167-7221

Ratmanee Chanabun https://orcid.org/0000-0003-4584-2773

Arthit Pholyotha https://orcid.org/0000-0001-6677-1164

Pongpun Prasankok https://orcid.org/0000-0001-5967-223X

Teerapong Seesamut https://orcid.org/0009-0003-9589-8641

Warut Siriwut https://orcid.org/0000-0001-6760-9724

Ruttapon Srisonchai https://orcid.org/0000-0002-7142-0999

Chirasak Sutcharit https://orcid.org/0000-0001-7670-9540

Somsak Panha https://orcid.org/0000-0002-4431-2458

### Data availability

All of the data that support the findings of this study are available in the main text.
